# Copper Forms a PPII Helix-Like Structure with the Catalytic Domains of Bacterial Zinc Metalloproteases

**DOI:** 10.1021/acs.inorgchem.3c02391

**Published:** 2023-11-01

**Authors:** Paulina Potok, Arian Kola, Daniela Valensin, Merce Capdevila, Sławomir Potocki

**Affiliations:** † Faculty of Chemistry, University of Wroclaw, 50-383 Wroclaw, Poland; ‡ Department of Biotechnology, Chemistry and Pharmacy, University of Siena, Via Aldo Moro 2, 53100 Siena, Italy; § Departament de Química, Universitat Autònoma de Barcelona, 08193 Cerdanyola del Vallès, Spain

## Abstract

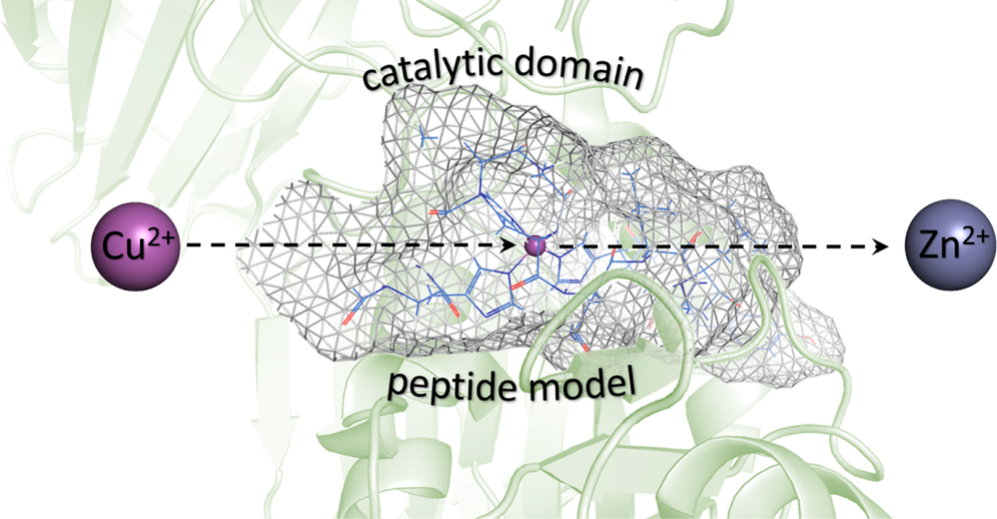

The rapid spread of antibiotic-resistant bacteria continuously raises concerns about the future ineffectiveness of current antimicrobial treatments against infectious diseases. To address this problem, new therapeutic strategies and antimicrobial drugs with unique modes of action are urgently needed. Inhibition of metalloproteases, bacterial virulence factors, is a promising target for the development of antibacterial treatments. In this study, the interaction among Zn(II), Cu(II), and the metal-binding domains of two metalloproteases, AprA (*Pseudomonas aureginosa*) and CpaA (*Acinetobacter baumanii*), was investigated. The objective was to determine the coordination sphere of Zn(II) with a peptide model of two zinc-dependent metalloproteases. Additionally, the study explored the formation of Cu(II) complexes with the domains, as Cu(II) has been shown to inhibit metalloproteases. The third aim was to understand the role of nonbinding amino acids in stabilizing the metal complexes formed by these proteases. This work identified specific coordination patterns (HExxHxxxxxH) for both Zn(II) and Cu(II) complexes, with AprA and CpaA exhibiting a higher affinity for Cu(II) compared to Zn(II). The study also found that the CpaA domain has greater stability for both Zn(II) and Cu(II) complexes compared to AprA. The nonbinding amino acids of CpaA surrounding the metal ion contribute to the increased thermodynamic stability of the metal–peptide complex through various intramolecular interactions. These interactions can also influence the secondary structures of the peptides. The presence of certain amino acids, such as tyrosine, arginine, and glutamic acid, and their interactions contribute to the stability and, only in the case of Cu(II) complexes, the formation of a rare protein structure called a left-handed polyproline II helix (PPII), which is known to play a role in the stability and function of various proteins. These findings provide valuable insights into the coordination chemistry of bacterial metalloproteases and expand our understanding of potential mechanisms for inhibiting these enzymes.

## Abstract 2

Metalloproteases (MPs) are a class of zinc-dependent enzymes that play a crucial role in bacterial infection. This study investigated the interactions among Zn(II), Cu(II), and the metal-binding regions of MPs from two multidrug-resistant bacteria. Copper can displace zinc in the catalytic sites of both MPs. Moreover, Cu(II) forms a rare left-handed polyproline II helix structure important for the stability and function of various proteins. The study provides insight into the potential additional mechanisms of MP inhibition.

## Introduction

Proteases (also known as proteinases, peptidases, and proteolytic enzymes) are essential to the existence of all kinds of living organisms at every stage of life.^1,2^ They are crucial in many biological proteolytic processes, including enzyme digestion and degradation, as well as blood clotting, immune defense, and the remodeling of cells and tissues.^2−6^ The mechanism of action is based on cleaving peptide bonds in proteins, which leads to the hydrolysis of the protein into smaller peptide fragments or individual amino acids.^7,8^

Proteolytic enzymes can be classified as serine, cysteine, aspartic, or metalloproteases based on the catalytic elements.^8,9^ Metalloproteases (MPs) are a diverse class of proteases that complex with a metal ion and polarize water molecules to perform the hydrolytic reaction.^7,10,11^ The MEROPS database provides one widely accepted classification of MPs, which classifies homologous sets of proteases and protein inhibitors into protein species, clans, and families based on evolutionary distances and sequence similarity.^12^ MPs are produced by all types of microorganisms, including both nonpathogenic and pathogenic bacteria or fungi. For most bacteria, MPs affect the physiology and biochemistry of the organism.^13^ However, when pathogenic microorganisms, especially opportunistic pathogens, produce these enzymes, MPs act as virulence factors that play a significant role in bacterial pathogenicity.^14−16^ Furthermore, many microorganisms require MPs to develop the capacity to infect their hosts and spread disease.^10,15^ In recent years, there has been increasing interest in the role of bacterial metalloproteases in human health and disease, with ongoing research focused on understanding their function and regulation.^17^ The rapid growth of multidrug-resistant bacterial species has compelled the World Health Organization to warn about a post-antibiotic era in which existing antimicrobial therapeutic strategies would be mostly ineffective against infectious pathogens.^18,19^ Therefore, it is crucial to identify new therapeutic targets and create antimicrobial drugs with atypical modes of action. Given the recent success of several protease modulators in the treatment of a variety of diseases, including the regulation of blood glucose levels (dipeptidyl peptidase 4)^20^ or HIV therapy,^21^ targeting bacterial metalloproteases essential to virulence represents promising therapeutic strategy for the next generation of antibacterial treatments. The biological role of potential protease-targeting drugs would be based on protease inhibition, which in the case of metalloproteases could be aimed at the catalytically active zinc-binding domain. However, additional knowledge on the structure and mechanism of action of metalloproteases and the stability of complexes formed with metals is required.^22^

In this work, the specificity of interactions between the metal ion and the active domains of two metalloproteases, AprA and CpaA, were analyzed (Table 1).^22^ AprA is an alkaline protease that is secreted by *Pseudomonas aureginosa* and belongs to the serralysin family of zinc-dependent MPs.^23^ Proteins belonging to the serralysin family are widespread virulence factors in Gram-negative bacteria such as *Serratia* and *Erwinia* species. AprA possesses a signal peptide, an N-terminal catalytic domain responsible for binding zinc ions and substrates, and a C-terminal domain. The active site located in the catalytic domain is characteristic of all MP zinc-binding motifs, HExxHxxGxxH. As a virulence factor of *P. aeruginosa*, AprA is produced in cases of cystic fibrosis, bacteremia, keratitis, and otitis media. Its function is to hydrolyze many biologically important proteins of the host’s immune system.^24,25^ The metalloprotease CpaA, being a member of the M72 protease family, is one of the main virulence factors secreted by the type II secretion system of *Acinetobacter baumannii*.^26,27^ Interestingly, the CpaA mutant was less virulent in both an invertebrate and a mouse model of pneumonia.^28^ CpaA contains the signal peptide near the N-terminal site and the C-terminal conserved proteolytic active site with the characteristic HExxHxxGxxH sequence.^29^ Similar to the primary sequence diversity of MPs, the three-dimensional structures, location of the catalytic domain, and size of AprA, CpaA, and other members of MPs also vary significantly.^10^ Although variations exist among MPs, they all share a common feature conserved across this enzyme class: a catalytic Zn(II) ion in the active site with a characteristic binding motif. Considering that deprivation of the catalytic Zn(II) by a chelator can lead to loss of the activity of MPs, finding efficient inhibitors targeting the conserved catalytic sites of MPs remains an important field of research.^10^ In order to provide information about the binding mode, structure, and thermodynamics of the interactions between the catalytic sites of the chosen MPs and the essential site for the Zn(II) activity, we decided to design a peptide model derived from active sites of AprA and CpaA.

**Table 1:**
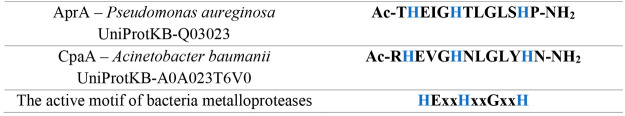
Amino Acid Sequences of the Examined Peptides and the Conserved Active Motif of the Metalloproteases

The main objective of the study was to identify metal binding sites in the examined protein regions, with a particular focus on understanding the role of nonbinding amino acids (e.g., arginine and tyrosine) in the stabilization of the formed metal complexes. The nonbinding amino acids surrounding the coordinated metal ion can contribute to the greater thermodynamic stability of the metal complex through various intramolecular interactions, including electrostatic interactions, hydrogen bonding, and van der Waals forces.^30−33^ These interactions slow the hydrolysis rate of metal–peptide bonds and consequently increase the stability of the complex. Moreover, the local interactions between amino acids determine the folding of the peptide chain into specific secondary structures, such as α-helices, β-sheets, or even uncommon left-handed polyproline II helices (PPII), which also influence the thermodynamic properties of the complex. Here we present which amino acids affect the stability of formed metal-peptide complexes and how the noncovalent interactions may influence the secondary structure of analyzed peptides. In addition, since MPs with the **H**Exx**H** binding motif can be inhibited by Cu(I) and Cu(II),^34^ the second part of this work was devoted to investigating the interaction of copper complexes with the selected domains. Due to the different coordination properties of Cu(II) and Zn(II) in biological systems,^35,36^ the substitution of the catalytic zinc with copper in a MP can interfere with its enzymatic activity.^37^ This is a potential starting point for the development of metal-based inhibitors of bacterial MPs.

## Experimental Section

### Peptide Synthesis and Purification

The peptides were obtained from KareBayTM Biochem, Inc. with a verified purity (98%) and were used as received. The identity of the peptide was further confirmed by mass spectrometry, and its purity was determined through the Gran method during potentiometric titrations.^38^ Metal ion solutions were prepared using Zn(ClO_4_)_2_, Cu(ClO_4_)_2_, and POCh (HPLC grade). The concentrations of the stock solutions were regularly verified via ICP-MS. Solutions of peptides were prepared using 4 × 10^–3^ mol·dm^–3^ HClO_4_ (Merck), and the ionic strength was adjusted to 0.1 mol·dm^–3^ through the addition of NaClO_4_ (Merck).

### Mass Spectrometric Measurements

The mass spectra were recorded for peptide and metal ion mixtures dissolved in a MeOH/H_2_O (1:1) solution with a 1:1 molar ratio. The ligand concentration was 1 × 10^–4^ M. The spectrometer measured Zn(II) and Cu(II) complexes with peptides in the positive mass-to-charge ratio (*m*/*z*) range of 300–1000. The mass spectra were acquired by means of a Bruker MicrOTOF-Q spectrometer (Bruker Daltonik, located in Bremen, Germany) with an Apollo II electrospray ionization source featuring an ion funnel. To calibrate the instrument, a Tunemix mixture (Bruker Daltonik, Germany) in the quadratic regression mode was used. The following parameters were applied: scan range of 300–1000 *m*/*z*, dry nitrogen gas, ion energy of 5 eV, and operating temperature of 180 °C. The capillary voltage was adjusted to achieve the highest signal-to-noise ratio, reaching 4800 V. The data were analyzed through the Bruker Compass DataAnalysis 4.0 software.

### Potentiometric Measurements

The stability constants of the proton and Zn(II) and Cu(II) complexes with peptides were determined via pH-metric titration curves. Measurements were conducted at 298 K, with a pH range of 2.5–11 and an ionic strength of 0.1 NaClO_4_. The total volume of the sample was 2.5 cm^3^. The experiment utilized a Dosimat 665 Methrom titrator, which was connected to a Methrom 691 pH meter. The pH electrode used was an InLab Semi-Micro instrument from Mettler Toledo. The thermostabilized cell glass was equipped with a microburet delivery tube, a magnetic stirring system, and an inlet–outlet tube for argon. The solutions were protected from carbonates by carrying out analysis under an argon atmosphere. A 0.1 M solution of carbonate-free NaOH was employed for the titration. The electrodes were calibrated each day for the hydrogen concentration through titration of HClO_4_ with NaOH. To establish the purity and exact concentrations of the ligand solutions, the Gran method was employed. The ligand (peptide) concentration was 0.5 × 10^–3^ mol·dm^–3^. In metal complex titrations, a molar ratio of 1.0:1.1 (metal/ligand) was used.

Stability constant calculations were performed using the HYPERQUAD 2006 software.^39^

Reported log β values refer to the overall equilibria.
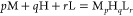
1
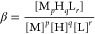
2log *K*_step_ values refer to the protonation process (charges are omitted; *p* might also be 0).
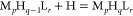
3HYPERQUAD 2006 software was used to calculate the standard deviations, which indicate random errors only. The calculations utilized constants for hydrolytic Zn(II) species.^40^ The HYSS program was used to create the competition and speciation diagrams.^41^

### Spectroscopic studies

Absorption spectra were recorded in the 800–250 nm range at 298 K using a Cary 300 Bio spectrophotometer with a total volume of 2.8 cm^3^. The peptide concentration was 0.5 × 10^–3^ mol·dm^–3^, and the optical length was measured to be 1 cm. To characterize the different species present in solution, we compared the observed wavelength of maximum absorption in the visible spectra at a specific pH value with the λ_max_ value obtained from literature.^42−44^ For this, we selected the conditions under which a particular species attains its maximum formation in solution. CD spectra were recorded using a Jasco J-1500 CD spectrometer within the 800–200 nm range. The data were then processed with Origin 9.0 software. EPR spectra were obtained using a Bruker ELEXSYS E500 CW-EPR spectrometer operating at X-band frequency (9.5 GHz) and fitted with an ER 036 NMR Teslameter and an E41 FC frequency counter. Solutions of peptides were prepared using 4 × 10^–3^ mol·dm^–3^ HClO_4_ with an ionic strength of 0.1 M (NaClO_4_). The concentration of Cu(II) was 1 × 10^–3^ M, with the molar ratio set at 1.0:1.1 (metal/ligand). The pH was regulated with suitable quantities of HCl and NaOH solutions. Ethylene glycol (25%) was employed as a cryoprotectant for the EPR measurements. The EPR parameters were examined by computer using WIN-EPR SIMFONIA software, version 1.2 (Bruker). NMR experiments were conducted using a 600 MHz Bruker Advance spectrometer equipped with a sensitive enhancement improvement (SEI) probe. All the experiments were performed at 298 K. The samples were prepared using a mixture of 90% H_2_O and 10% D_2_O (Merck). The analyzed complexes were prepared by adding a small volume of Zn(ClO_4_)_2_ stock solution to an acidic peptide solution containing 0.8 × 10^–3^ mol·dm^–3^ ligand (pH 5) and subsequently increasing the pH to 7.0. To suppress the residual water signal, a selective 2 ms long square pulse was applied to water using the excitation sculpting pulse program.^45^ Proton resonance was assigned using 2D ^1^H–^1^H total correlation spectroscopy (TOCSY) and nuclear Overhauser effect spectroscopy (NOESY) experiments with standard pulse sequences. The resulting NMR data underwent processing and analysis using the TopSpin 3.6.4 (Bruker) program.

## Results and Discussion

In this work, we describe the thermodynamic and structural properties of Zn(II), and Cu(II) complexes with the metal-binding sites of two metalloproteases, Ac-THEIGHTLGLSHP-NH_2_ (AprA), and Ac-RHEVGHNLGLYHN-NH_2_ (CpaA). The precisely chosen regions are essential for the bioactivity of the examined virulence factors. The complexes were investigated using various techniques, including NMR, CD, UV–vis, mass spectrometry, and potentiometry. Potentiometric titrations were employed to determine the stability constants and pH-dependent species distribution diagrams. The stoichiometry of the interactions was determined via mass spectrometry, whereas the binding modes and geometry of Cu(II) species in the solution were elucidated through a combined approach of UV–vis and CD results. The NMR spectra recorded with and without zinc ions indicated precisely the amino acids involved in metal coordination. The combination of all of the methods used allowed for the elucidation of the thermodynamic parameters and geometry of the formed metal complexes, as well as the coordination properties of the ligands.

### Ligand Protonation

The protonation constants for the peptides are presented in Table 2, while Table 3 contains the complex formation constants along with the most probable coordination environment for each species formed. The two investigated peptides were protected at their N-terminus by acetylation and at their C-terminus by amidation; therefore, their acid–base behavior is determined by the properties of the amino acid side chains.

**Table 2:** Protonation Constants of the Examined Peptides at 298 K in Aqueous Solution^a^

AprA (Ac-THEIGHTLGLSHP-NH_2_)			CpaA (Ac-RHEVGHNLGLYHN-NH_2_)		
species	log β	p*K*_a_	species	log β	p*K*_a_
HL	7.48(8)	7.48 (His)	HL	9.86(2)	9.86 (Tyr)
H_2_L	13.84(6)	6.36 (His)	H_2_L	16.83(4)	6.97 (His)
H_3_L	20.05(7)	6.21 (His)	H_3_L	23.23(3)	6.40 (His)
H_4_L	24.07(8)	4.02 (Glu)	H_4_L	28.98(3)	5.75 (His)
			H_5_L	32.42(3)	3.44 (Glu)

a The ligand L is a fully deprotonated peptide.

**Table 3:** Equilibrium Constants and Proposed Coordination Modes for Zn(II) and Cu(II) Complexes in Aqueous Solutions at a Temperature of 298 K^a^

AprA (Ac-THEIGHTLGLSHP-NH_2_)				CpaA (Ac-RHEVGHNLGLYHN-NH_2_)			
species	log β	p*K*_a_	coordination	species	log β	p*K*_a_	coordination
Cu(II) complexes							
CuH_2_L	19.59(4)		N_im_, COO^–^	CuH_2_L	22.34(4)		2N_im_, COO^–^
				CuHL	17.08(2)	5.26	3N_im_, COO^–^
CuL	8.94(4)		3N_im,_ COO^–^	CuL	10.47(5)	6.61	3N_im,_ 1N^–^
CuH_–1_L	1.55(8)	7.39	3N_im_, 1N^–^	CuH_–1_L	3.36(4)	7.11	2N_im_, 2N^–^
CuH_–2_L	–6.33(6)	7.88	2N_im_, 2N^–^	CuH_–2_L	–5.58(6)	8.94	2N_im_, 2N^–^
CuH_–3_L	–15.58(7)	9.25	1N_im_, 3N^–^	CuH_–3_L	–15.79(5)	10.21	1N_im_, 3N^–^
Zn(II) complexes							
ZnHL	10.83(7)		2N_im_, COO^–^	ZnHL	14.27(5)		3N_im_, COO^–^
ZnL	4.56(3)	6.27	3N_im_, COO^–^	ZnL	7.56(4)	6.71	3N_im_, O_H2O_
ZnH_–1_L	–3.63(4)	8.19	3N_im_, O_H2O_	ZnH_–1_L	–1.48(6)	9.04	3N_im,_ O_H2O_
ZnH_–2_L	–13.00(4)	9.37	3N_im_, O_H2O_	ZnH_–2_L	–12.17(8)	10.69	3N_im_, O_H2O_

a The ligand L is a fully deprotonated peptide that binds metal ions. Standard deviations are reported in parentheses on the last significant digit.

The Ac-THEIGHTLGLSHP-NH_2_ peptide behaves as an LH_4_ acid, with the deprotonating groups corresponding to the glutamic acid side chain carboxylate (p*K*_a_ = 4.02) and three histidine imidazoles with p*K*_a_ values 6.21, 6.36, and 7.48, respectively. In the case of Ac-RHEVGHNLGLYHN-NH_2,_ the five detected protonation constants correspond, respectively, to the carboxylic side chain group of glutamic acid (p*K*_a_ = 3.44), three imidazole groups of histidine residues (p*K*_a_ = 5.75, 6.40, and 6.97), and the side chain of the tyrosine (p*K*_a_ = 9.86, the most basic one).

### Copper Complexes

#### AprA Metal-Binding Domain

**Figure 1:**
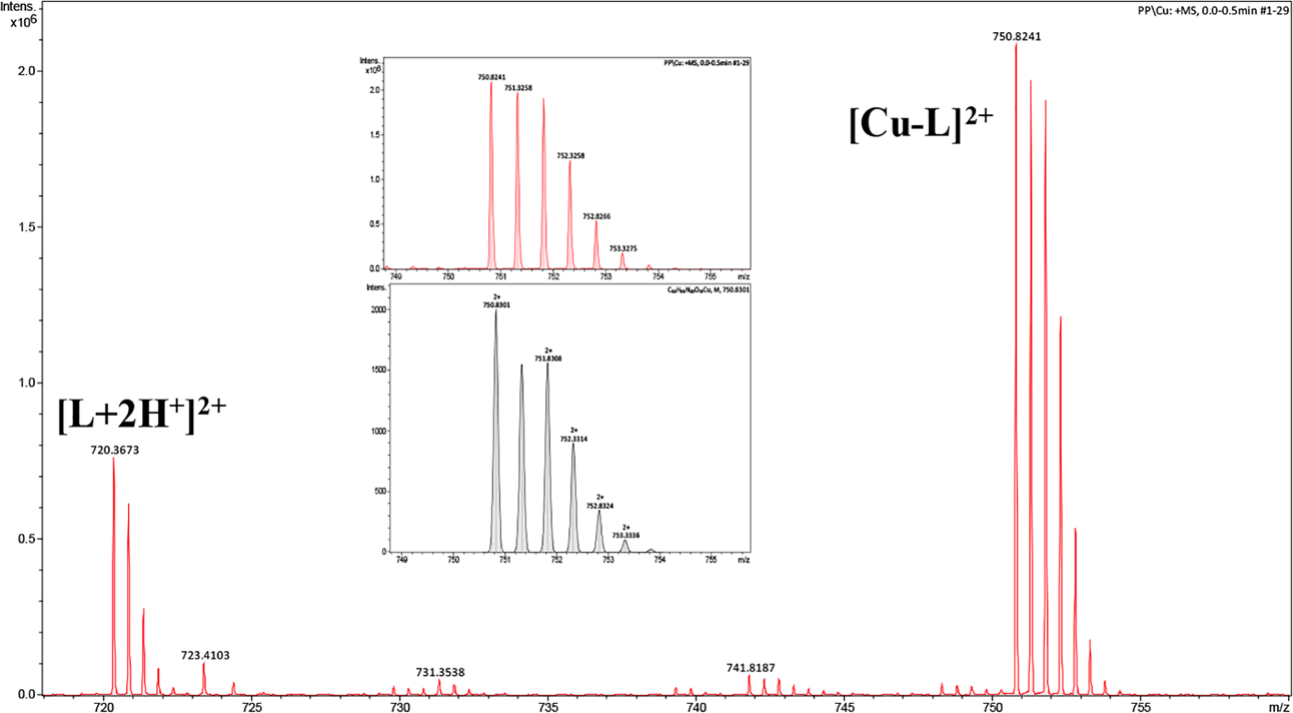
ESI-MS spectrum of a system composed of the AprA (Ac-THEIGHTLGLSHP-NH_2_) ligand (L) and Cu(II) ions in the range of *m*/*z* 710–765 at pH 7.0 (1:1 M:L). In the middle, the simulated and experimental isotopic distribution spectra with a peak at *m*/*z* 750.82 are presented.

The mass spectrum of the Cu(II)–ligand system with the AprA metal-binding domain is shown in Figure 1. In the spectrum, we can observe the [L + 2H^+^]^2+^ (*m*/*z* 720.37; *z* = 2+) signal corresponding to the free ligand ion as well as the [CuL]^2+^ (*m*/*z* 750.82; *z* = 2+) signal corresponding to the mononuclear Cu(II)–ligand complex.

**Figure 2:**
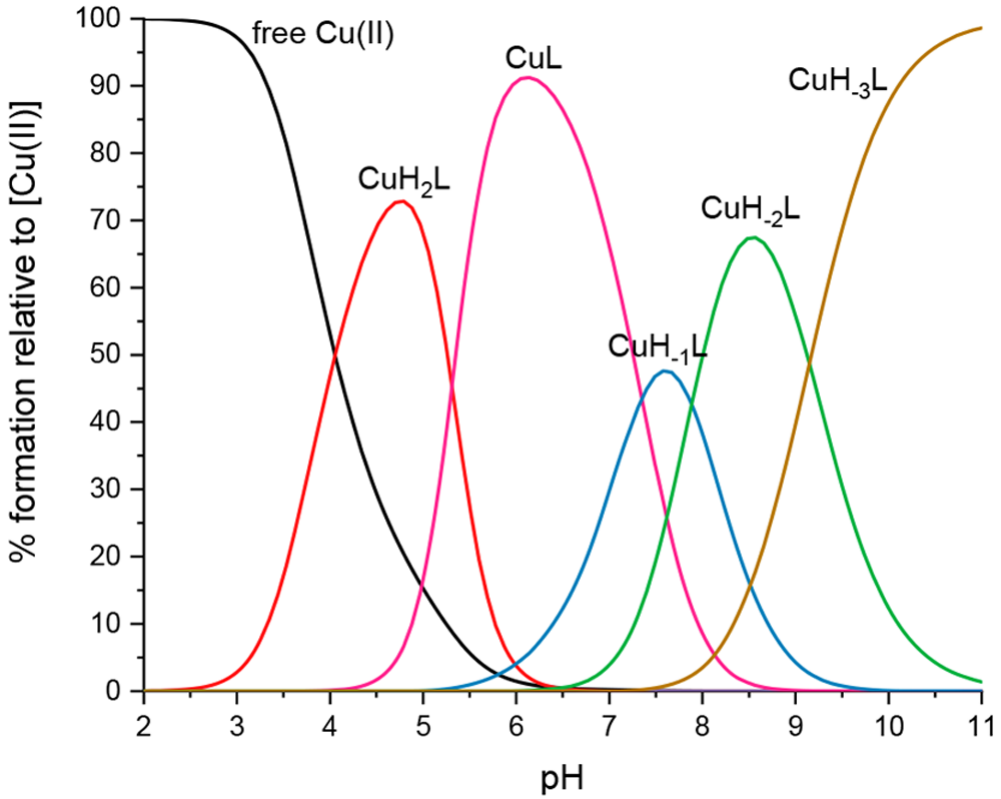
Species distribution diagram for Cu(II)-AprA (Ac-THEIGHTLGLSHP-NH_2_) complexes at a 1.0:1.1 Cu (II)/peptide ratio in an aqueous solution.

Copper starts to interact with the Ac-THEIGHTLGLSHP-NH_2_ peptide at a pH lower than 3, when the first detected complex (CuH_2_L) forms (Figure 2).

**Figure 3:**
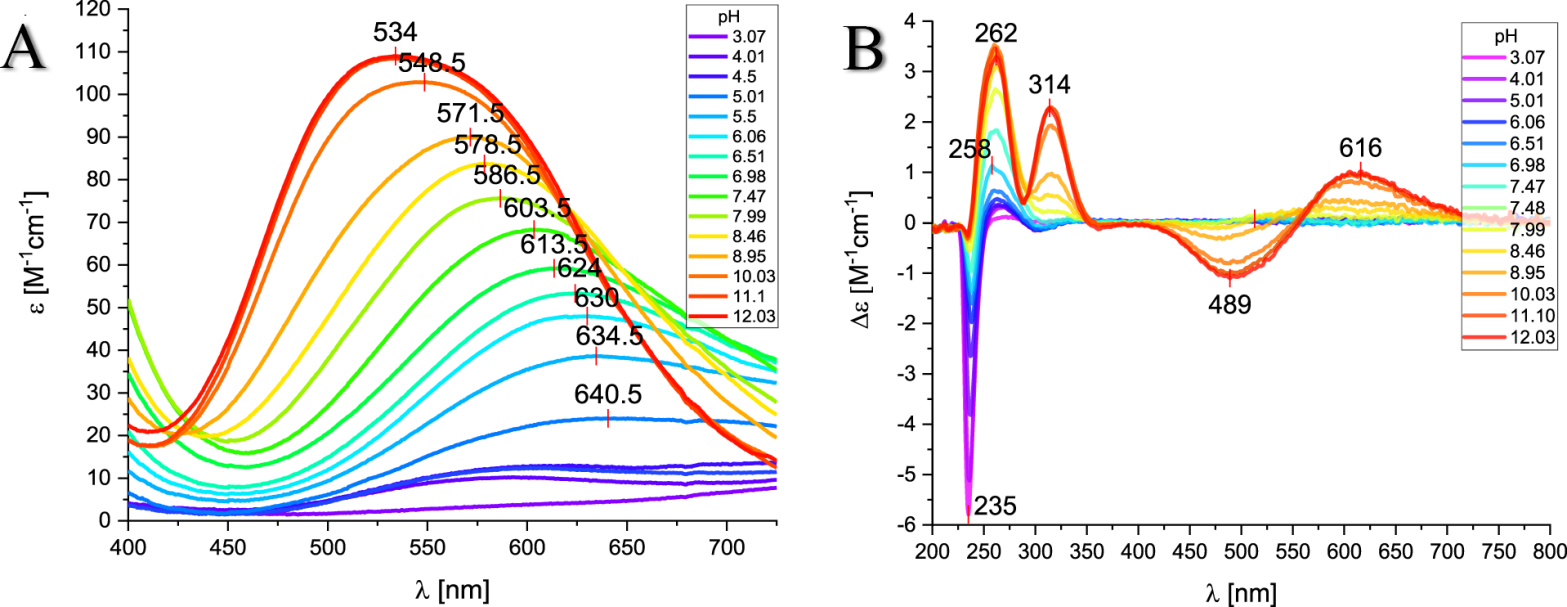
(A) Visible absorption spectra and (B) CD spectra of Cu(II)-AprA (Ac-THEIGHTLGLSHP-NH_2_) at different pH values and a 1.0:1.1 Cu(II)/peptide ratio. The wavelength of the maximum absorption is reported for each spectrum.

The stoichiometry of this species indicates that Cu(II) binds imidazole nitrogen from one histidine and the carboxylate group of the glutamic acid (coordination mode N_im_, COO^–^). This species is rapidly replaced by the most abundant form of copper complexes, CuL, which is formed by the deprotonation and coordination of the next two histidines (Figure 2). The glutamic acid residue most likely still completes the coordination sphere (3N_im,_ COO^–^). This species dominates around pH 6.2, at which parameters obtained by spectroscopic methods supported the assumed binding mode, particularly the position of the d–d band of the UV–vis spectra at pH 6.06 (λ_max_ = 630 nm, expected for 3N_im_ binding) (Figure 3A), the EPR spectra at pH 6.05 (*A*∥ = 162.5, *g*∥ = 2.28) (Figure S1, Table S1), and the characteristics of the charge transfer band of N_im_ → Cu(II) detected in the CD spectra at this pH (λ_max_ = 258 nm) (Figure 3B).^44,46^ The interaction of copper ions with the peptide backbone gradually occurs above a pH of 6. With increasing pH values, the nitrogen in imidazole is replaced by amide nitrogen, leading to the coordination mode changing from (3N_im,_ COO^–^) to (3N_im_, 1N^–^) for CuH_–1_L, (2N_im_, 2N^–^) for CuH_–2_L, and (1N_im_, 3N^–^) for CuH_–3_L (Figure 2). This hypothesis is confirmed by the spectroscopic results shown in Figure 3A and B: the d–d band in the visible spectra blueshifts from λ_max_ = 578.5 (3N_im_, 1N^–^) at pH 8.46 to 534 nm (1N_im_, 3N^–^) at pH 11.1, and the charge transfer transitions change in the CD spectra, from N_im_ → Cu(II) at 258 nm to the increased intensity of the band for N^–^ → Cu(II) at 262 nm for alkaline pH and the characteristic square planar geometry bands at 489 and 616 nm. Moreover, the EPR parameters (Table S1) obtained at pH 10 (A∥ = 201.70 g∥ = 2.19) confirm the binding mode with four nitrogens.^44,46^ Considering the impact of copper ions on the structure of the analyzed peptide, at pH 9.05–11.14 in the far-UV CD spectrum of the Cu(II)-AprA complex, two negative peaks near 200 and 225 nm are observed together with the positive peak at 183 nm. This result suggests the formation of the helical structure of the peptide complex, which may have an impact on the stabilization of the complex (Figure S2).^42,47^

#### CpaA Metal-Binding Domain

**Figure 4:**
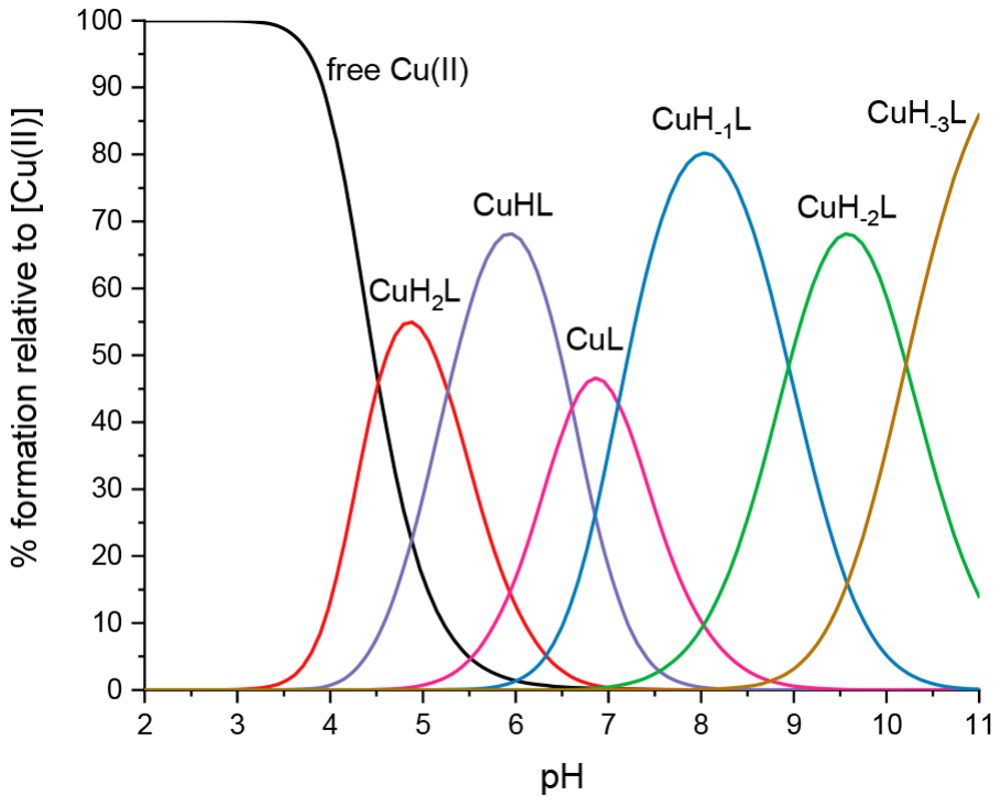
Species distribution diagram for Cu(II)-CpaA (Ac-RHEVGHNLGLYHN-NH_2_) complexes at a 1.0:1.1 Cu(II)/peptide ratio in an aqueous solution.

The ESI-MS spectrum (Figure S3) shows signals of the free ligand ions ([L + 2H^+^]^2+^, *m*/*z* 793.90; *z* = 2+) and an equimolar complex with Cu(II) ions ([CuL]^2+^, *m*/*z* 824.86; *z* = 2+), indicating that only mononuclear species are formed. According to the calculations based on potentiometric titrations of Cu(II)-CpaAin 4 mM HClO_4_ (0.1 M NaClO_4_), six mononuclear forms were detected: CuH_2_L, CuHL, CuL, CuH-_1_L, CuH-_2_L, and CuH-_3_L (Figure 4 and Table 3).

**Figure 5:**
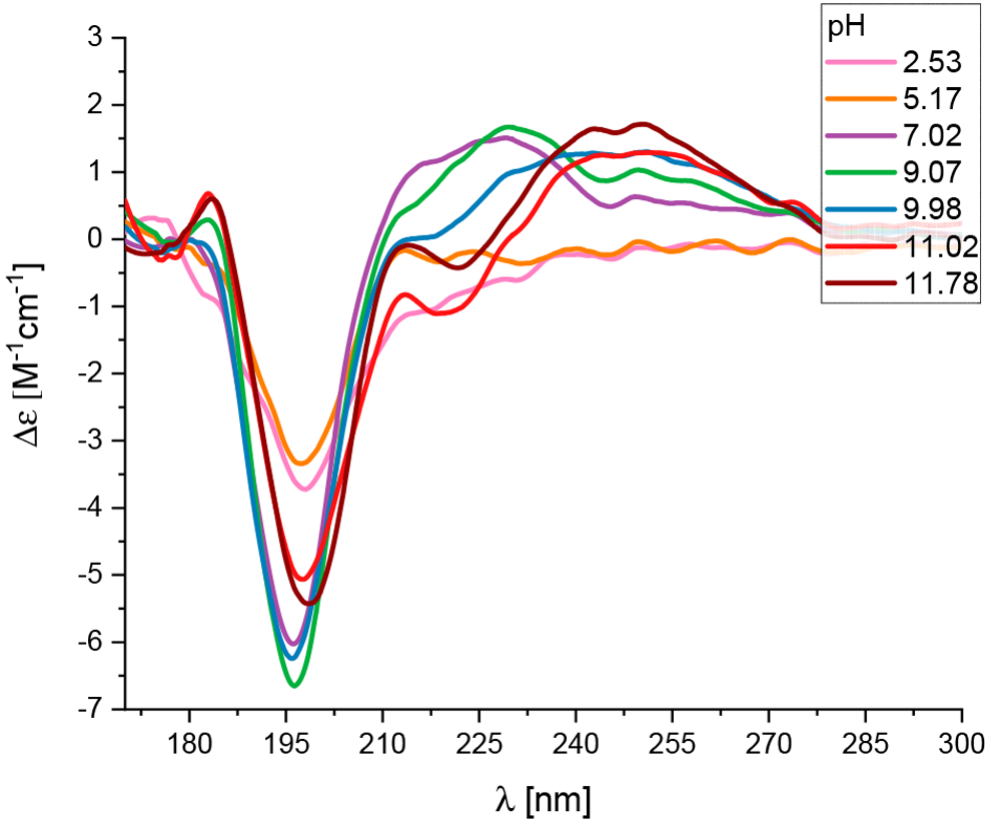
Far-UV CD spectra for the Cu(II)-CpaA (Ac-RHEVGHNLGLYHN-NH_2_) complex at different pH values and a 1.0:1.1 Cu(II)/peptide ratio in an aqueous solution.

The copper coordination starts at approximately pH 3 (Figure 4), forming the CuH_2_L species. This corresponds to the deprotonation and coordination of the initial two histidine residues, with the potential involvement of one carboxylate group from glutamic acid (2N_im_, COO^–^ coordination mode) (Table 3). Subsequent loss of a proton leads to CuHL species, which reach their maximum amount at pH 6. At this stage, the third histidine residue coordinates with the metal ion. This result agrees with the EPR parameters for this pH (*A*∥ = 168.2, *g*∥ = 2.29, as expected for three nitrogens in the copper’s coordination sphere) (Table S2 and Figure S4).^44,46^ The (3N_im_, COO^–^) coordination mode is also supported by the UV–vis band λ_max_ = 635.5 nm (Figure S5A) and is characteristic of the charge transfer of N_im_ → Cu(II) detected in the CD spectra λ_max_ = 255 nm, which obtained its maximum of absorption at pH 8 (Figure S5B). The interaction of the copper ion with the nitrogen from the peptide backbone most likely begins above pH 6, when CuL and CuH_–1_L species start to form with the binding modes (3N_im_, 1N^–^) and (2N_im_, 2N^–^), respectively. In the obtained UV–vis and CD spectra, greater involvement of the peptide chiral centers (N^–^amide donor groups) is observed through the shifts of wavelength in both UV–vis and CD spectra at an alkaline pH (Figure S5A and B). The loss of the next proton results in the formation of CuH_–2_L species, with maximum formation at pH 9.5. Spectroscopic data do not evidence any significant change between pH 8 and 9, indicating that the CuH_–2_L species is related to the deprotonation of tyrosine, which is not involved in metal binding. However, in the potentiometric results, we observe the lowered p*K*_a_ value of tyrosine in the complex (p*K*_a_ = 8.94) in comparison to the p*K*_a_ of 9.86 in the free ligand (Table 3). During the binding of copper ions to the studied ligand, conformational changes occur (Figure 5) that create new weak interactions and a local microenvironment that may lower the p*K*_a_ of the tyrosine side chain. The tyrosine OH group’s ability to act as a hydrogen bond donor in this context is important. The p*K*_a_ of tyrosine is usually perturbed by the presence of neighboring charged amino acid residues,^48,49^ which is especially true when conformational changes occur. Additionally, the negative charge on the oxygen atom can stabilize the binding of cations, leading to a decrease in p*K*_a_.^50^ In the next form, the increased participation of amide nitrogen, which substitutes the imidazole nitrogen as donor groups, results in the (1N_im_, 3N^–^) binding mode for CuH_–3_L species found above pH 9 (Figure 4). The greater involvement of the amide nitrogen in the coordination sphere is confirmed by spectroscopic data; above pH 9 in the visible spectrum, the absorption blue shifts λ_max_ from 587 to 525 nm (Figure S5A). The formation of a metal coordination sphere composed of three deprotonated amide nitrogen atoms at pH 10 is further validated by the EPR parameters (*A*∥ = 201.7, *g*∥ = 2.19) (Table S2).^44,46^ Moreover, the new charge transfer transition CD band for N^–^ → Cu(II) at λ_max_ = 268 and 317 nm, along with the bands for the square planar complex geometry at λ_max_ = 497 and 637 nm, confirms the suggested coordination for alkaline pH (Figure S5B). Interestingly, between pH 7.00 and 9.00 (when the copper starts interacting with peptide backbone), the Cu(II)–peptide complex adopts the left-handed polyproline II helix (PPII) structure, which is supported by the far-UV CD spectrum (characteristic negative band at 195 nm and a positive band at 215 nm^51−53^) (Figure 5). As the name suggests, proline predominates in PPII helices; however, PPII can also be observed for polypeptides that do not contain any proline residues in the sequence. Moreover, positively charged residues, especially at the first position, together with leucine at the central positions of the peptide are strongly favored in this secondary structure.^54^ Arginine is a positively charged amino acid with a guanidinium side chain (−NH_3_^+^) that is highly polar and can participate in hydrogen bonding interactions with the surrounding solvent and other molecules. It was already described in the literature that electrostatic interactions stabilize the formation of the PPII structure.^54^ In the case of the Cu(II)–CpaA complex at pH 7, this interaction is observed between positively charged Arg-1 and the negative side chain of Glu-3.^53^ Neutralization of the guanidine group (−NH_3_^+^) of Arg-1, by raising the pH, induces structural changes of the Cu(II)-CpaA complex (Figure 5), confirming the assumed role of the electrostatic interactions in stabilizing the PPII helical structure. Moreover, Leu-8 and/or Leu-10, which are nonpolar amino acids with a relatively large hydrophobic side chain, also stabilize the PPII helix structure because of their unique side chain conformation. Their nonpolar side chains sterically favor the formation of trans-peptide bonds, which lead to the stable PPII helical conformation.^54^ This is a very uncommon structure that may have a significant impact on the stabilization of the metal complex.

### Zinc Complexes

#### AprA Metal-Binding Domain

**Figure 6:**
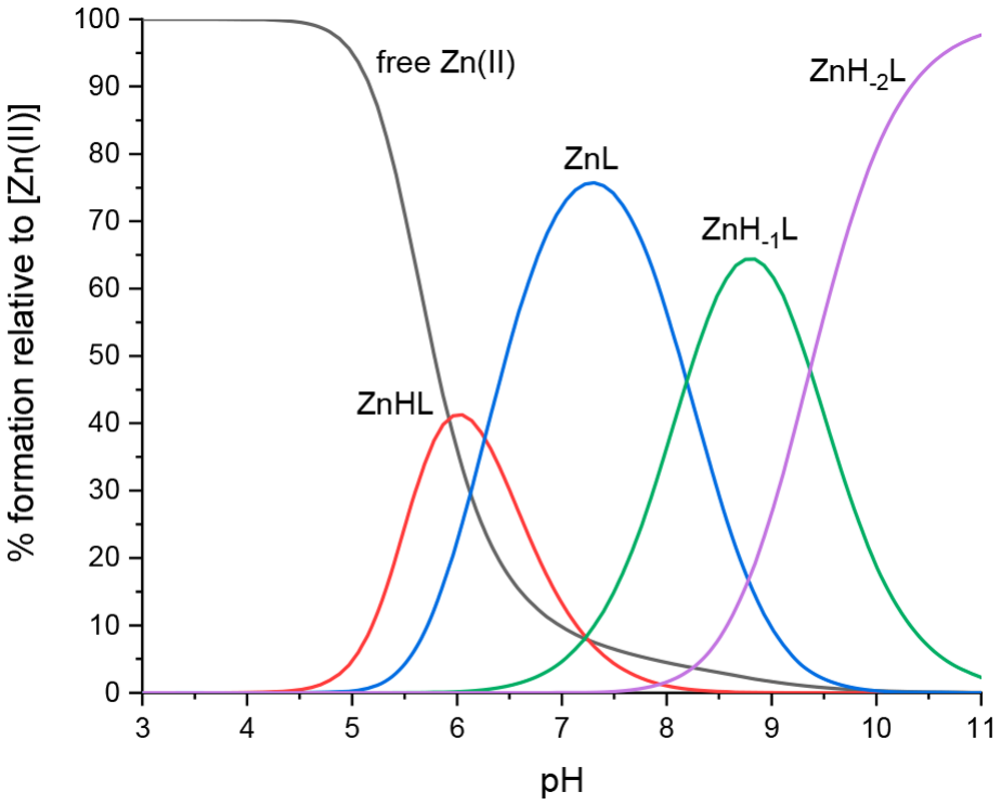
Species distribution diagram for Zn(II)-AprA (Ac-THEIGHTLGLSHP-NH_2_) complexes at a 1.0:1.1 Zn(II)/peptide ratio in an aqueous solution.

The mass spectrum of the Zn(II)-AprA system is presented in Figure S6. In the spectrum, we observe signals corresponding to the free ligand ion [L + 2H^+^]^2+^ (*m*/*z* 720.37; *z* = 2+) and to an equimolar complex with Zn(II) ions [ZnL]^2+^ (*m*/*z* 751.33; *z* = 2+). Using the potentiometric calculations based on the pH titrations of Zn(II)-Ac-THEIGHTLGLSHP-NH_2_ complexes in 4 mM HClO_4_ (0.1 M NaClO_4_), four Zn(II) complex species were identified: ZnHL, ZnL, ZnH__–1__L, and ZnH__–2__L (Figure 6 and Table 3).

The interaction between the AprA metal-binding domain peptide and Zn(II) starts at pH 4.5 with the ZnHL species via the coordination of two histidine residues along with the glutamic acid side chain (2N_im_, COO^–^) (Table 3). The most abundant form at psychological pH is ZnL, which forms at pH 5 and reaches its maximum concentration at a pH of about 7.25 (Figure 6). The p*K*_a_ of 6.26 shows a significant reduction in the complex compared to the p*K*_a_ of 7.48 for this residue in the free ligand. This suggests the presence of a third histidine side chain in the coordination sphere (3N_im_, COO^–^) (Table 3). The next two p*K*_a_ values (8.19, and 9.37) corresponding to the ZnH_–1_L, and ZnH_–2_L species, respectively, are most likely associated with the deprotonation of the water molecules. The far-UV circular dichroism (CD) spectra of the Zn-AprA complex at acidic pH show no clear tendency of the ligand to adopt an ordered secondary structure; however, when the pH increases, bands around 182, 198, and 229 nm suggest sharing of the helical structure from pH 7.09 (Figure S7).^47^ The α-helix structure is associated with the reorganization of electrostatic and hydrogen bond interactions; therefore, the pH-inducted structural formation may provide additional stabilization for the Zn-AprA complex at pH levels in the 7.09–11.26 range.^55^

#### CpaA Metal-Binding Domain

**Figure 7:**
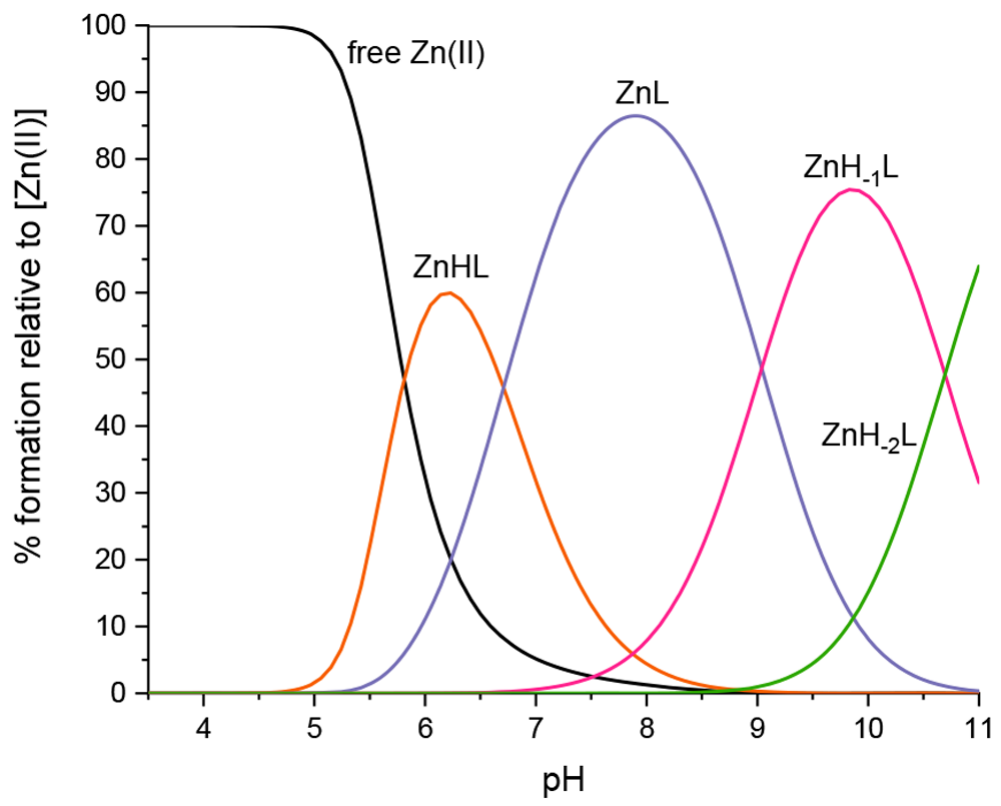
Species distribution diagram for Zn(II)-CpaA (Ac-RHEVGHNLGLYHN-NH_2_) complexes at a 1.0:1.1 Zn(II)/peptide ratio in an aqueous solution.

The ESI-MS results revealed that the Zn(II)-Ac-RHEVGHNLGLYHN-NH_2_ complex forms only mononuclear species. The spectrum shows signals corresponding to the free ligand ions ([L + 2H^+^]^2+^, *m*/*z* 793.90; *z* = 2+) and an equimolar Zn(II) complex ([ZnL]^2+^, *m*/*z* 824.86; *z* = 2+) (Figure S8). The potentiometric titrations of the Zn(II)-Ac-RHEVGHNLGLYHN-NH_2_ system in 4 mM HClO_4_ (0.1 M NaClO_4_) showed the existence of four complex forms at pH 2.5–11: ZnHL, ZnL, ZnH_–1_L, and ZnH_–2_L (Figure 7).

The first complex species detected is ZnHL, which initiates formation at pH 5 and reaches its maximum concentration around pH 6. These species involve all three histidine residues with the carboxyl group of glutamic acid in metal binding (coordination mode 3N_im_, COO^–^) (Table 3). The following identified complex is ZnL, formed at pH 5.5, with a maximum concentration at approximately pH 8 (Figure 7). The ZnL complex corresponds to the deprotonation of a water molecule bound to the central metal ion. Above pH 7, the ZnH_–1_L complex begins to form. It arises from the deprotonation of the tyrosine side chain, which is not involved in metal binding but has an impact on the stabilization of the formed zinc complex, as confirmed by NMR results (next section). ZnH_–2_L is the last detected complex, whose formation is most probably related to the deprotonation of the second water molecule. The secondary structure of the zinc complex at acidic pH exhibits a random coil conformation (confirmed by the characteristic negative band at 197 nm) (Figure S9). However, a higher content of the helical structure occurs from pH 7, which is evidenced by a decrease in the band at 197.1 nm and, simultaneously, the emergence of new peaks around 183, 198, and 225 nm.^47^ As we described in the previous domain complex, the α-helix structure may also have a stabilizing effect on the formed Zn(II)-CpaA complex due to intramolecular interactions.

### NMR Results

#### AprA and CpaA Free Ligands

The ^1^H NMR spectra of AprA and CpaA show the typical features of flexible and disordered peptides. At pH 7.4 and room temperature, several NH signals disappeared and NOESY spectra were characterized by weak and few NOE cross-peaks. NMR assignment of ^1^H signals was therefore obtained by combining the information obtained from the spectra recorded at acidic (pH 5) and physiological pH (7.0). The complete NMR assignments of both peptides at pH 7 are reported in Tables S3 and S4.

#### AprA and CpaA Zn(II) Complexes

**Figure 8:**
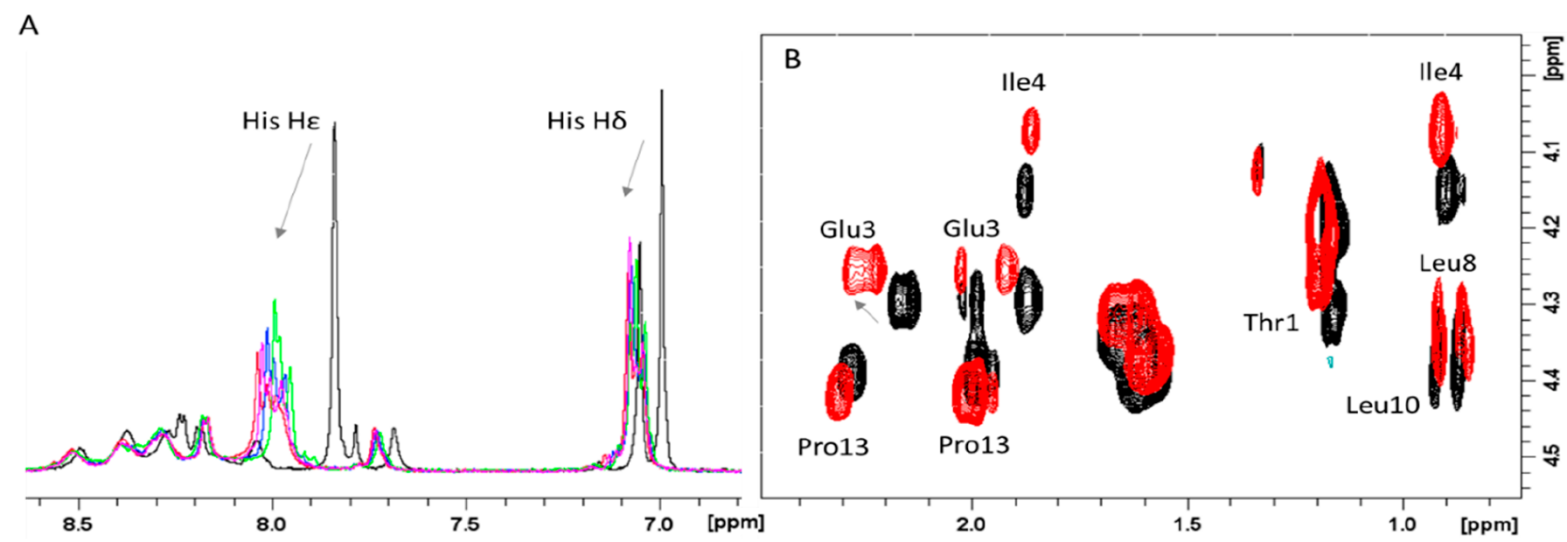
Superimposition of NMR spectra of 0.8 mM AprA (Ac-THEIGHTLGLSHP-NH_2_), pH 7.0. (A) ^1^H 1D spectra in the absence (black) and in the presence of 0.375 (green), 0.5 (blue), 0.625 (magenta), and 0.75 (red) equiv of Zn(II). (B) ^1^H–^1^H TOCSY spectra in the absence (black) and in the presence of 0.75 (red) equiv of Zn(II).

Upon the addition of Zn(II) to AprA solutions, we observed selective chemical shift variations on His aromatic protons, which were gradually shifted by increasing metal concentrations (Figure 8A). These effects strongly indicated the involvement of His imidazoles in Zn(II) binding, moreover the largest effects observed on Hε signals pointed out zinc coordination to His Nδ rather than Nε, the former being much closer to Hε if compared to Hδ.

**Figure 9:**
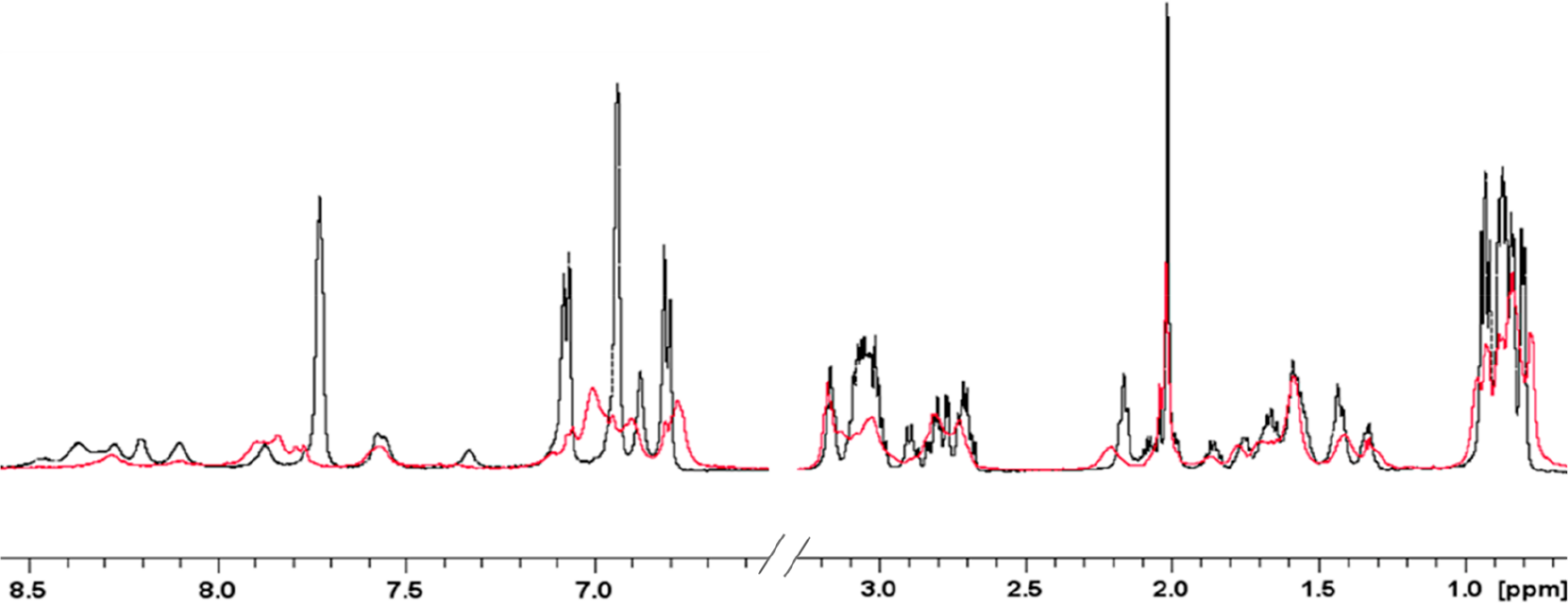
Superimposition of ^1^H 1D NMR spectra of 0.8 mM CpaA (Ac-RHEVGHNLGLYHN-NH_2_), pH 7.0, in the absence (black) and in the presence of 0.75 equiv of Zn(II).

**Figure 10:**
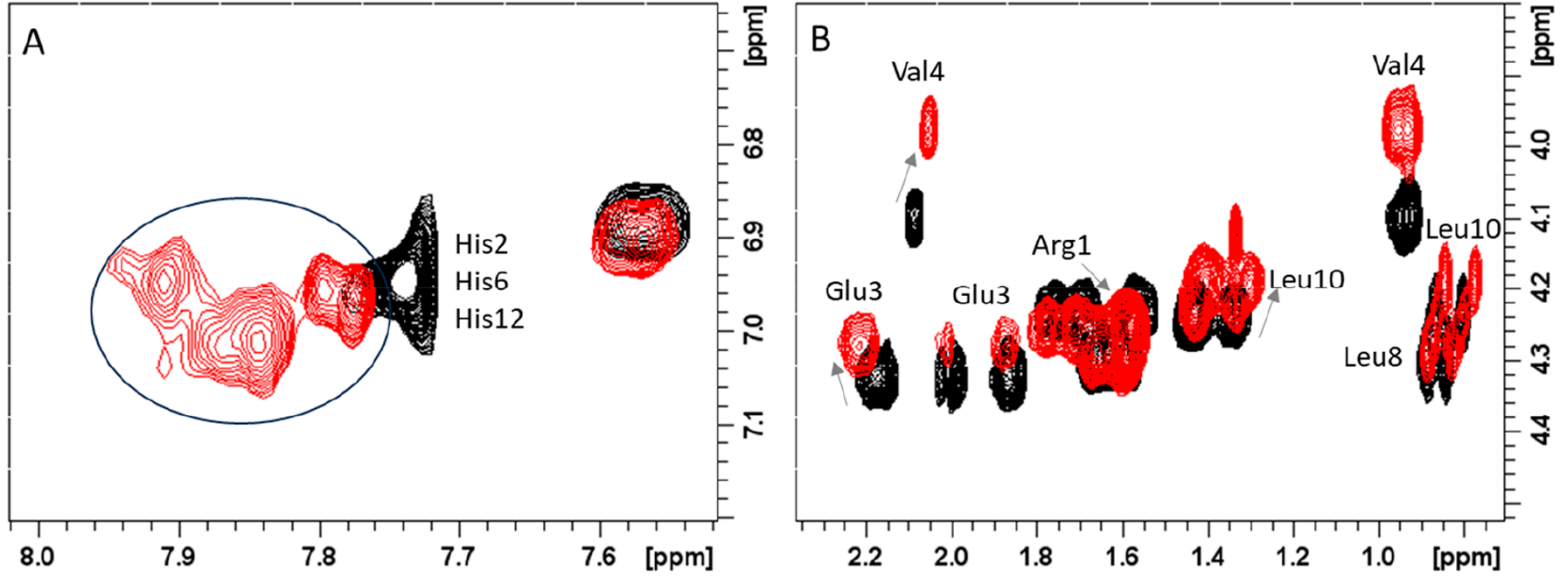
Superimposition of (A) aromatic and (B) aliphatic regions of ^1^H–^1^H TOCSY spectra of 0.8 mM CpaA (Ac-RHEVGHNLGLYHN-NH_2_), pH 7.0, in the absence (black) and in the presence of 0.75 (red) equiv of Zn(II).

The analysis of 2D spectra further revealed significant chemical shift variations on residues nearby the three His, such as Thr-1, Glu-3, Ile-4, Leu-8, Leu-10, and Pro-13 of AprA systems (Figure 8B). Among them, the changes on Hγ of Glu-3 support the involvement of Glu-3 carboxylate in the metal binding site. The overall chemical shift variations induced by the addition of 0.75 equiv of Zn(II) on AprA are shown in Table 4. Similar experiments were performed on CpaA-Zn(II) systems (Table 5). The obtained results revealed completely different NMR behavior. Upon Zn(II) addition, extensive line broadening was observed, with the aromatic and amide regions more being affected than the aliphatic one (Figure 9). However, the analysis of the 2D TOCSY experiments recorded in the absence and in the presence of 0.75 equiv of Zn(II) revealed selective chemical shift variations induced by the metal ion. As shown in Figure 10, the largest effects are exhibited by the three His, Arg-1, Glu-3, Val-4, and Leu-10.

**Table 4:** Zn(II)-Induced Chemical Shift Variations (δ_Zn_ – δ_apo_) of 0.8 mM AprA (Ac-THEIGHTLGLSHP-NH_2_) + Zn(II) at pH 7.0

residue	Hα	Hβ	Hγ	Hδ	Hε
Thr-1	–0.05	–0.01	0.03		
His-2	–0.06	–0.02/0.04		0.08	0.16
Glu-3	0	0.06/0.05	0.10		
Ile-4	–0.07	–0.01	0	0.02	
Gly-5	0.04				
His-6	–0.01	0.05/0.05		0.05	0.19
Thr-7	0.03	0.01	0.03		
Leu-8	–0.02	0.01	0	0	
Gly-9	–0.06				
Leu-10	–0.03	0.01	0.01	0/0.01	
Ser-11	–0.03	0.01			
His-12	0.02	0.06/0.04		0.03	0.19
Pro-13	0.03	0.03/0		0.01/0.06	

**Table 5:** Zn(II)-Induced Chemical Shift Variations (δ_Zn_ – δ_apo_) of 0.8 mM CpaA (Ac-RHEVGHNLGLYHN-NH_2_) at pH 7.0

residue	Hα	Hβ	Hγ	Hδ	Hε
Arg-1	0.04	0.03/0.04	0.03	0.02	
His-2^a^	0.02	0.02/0.01		0.08	0.11
Glu-3	0.02	0.01	0.03		
Val-4	–0.11	–0.03	0.02/0.01		
Gly-5	0				
His-6^a^	–0.02	0.01		0.09	0.12
Asn-7	–0.01	–0.02			
Leu-8	–0.01	0	0	0.00/0.02	
Gly-9	0				
Leu-10	0	0		0.00/0.04	
	–0.04	–0.03/–0.04		–0.03/-0.03	
Tyr-11	0	0		0.00	0.00
	–0.04	–0.09/–0.06		–0.07	–0.04
His-12^a^	–0.04	–0.02		0.03	0.17
Asn-13	0.04	0.04/0.05			

a These assignments can be exchanged.

**Figure 11:**
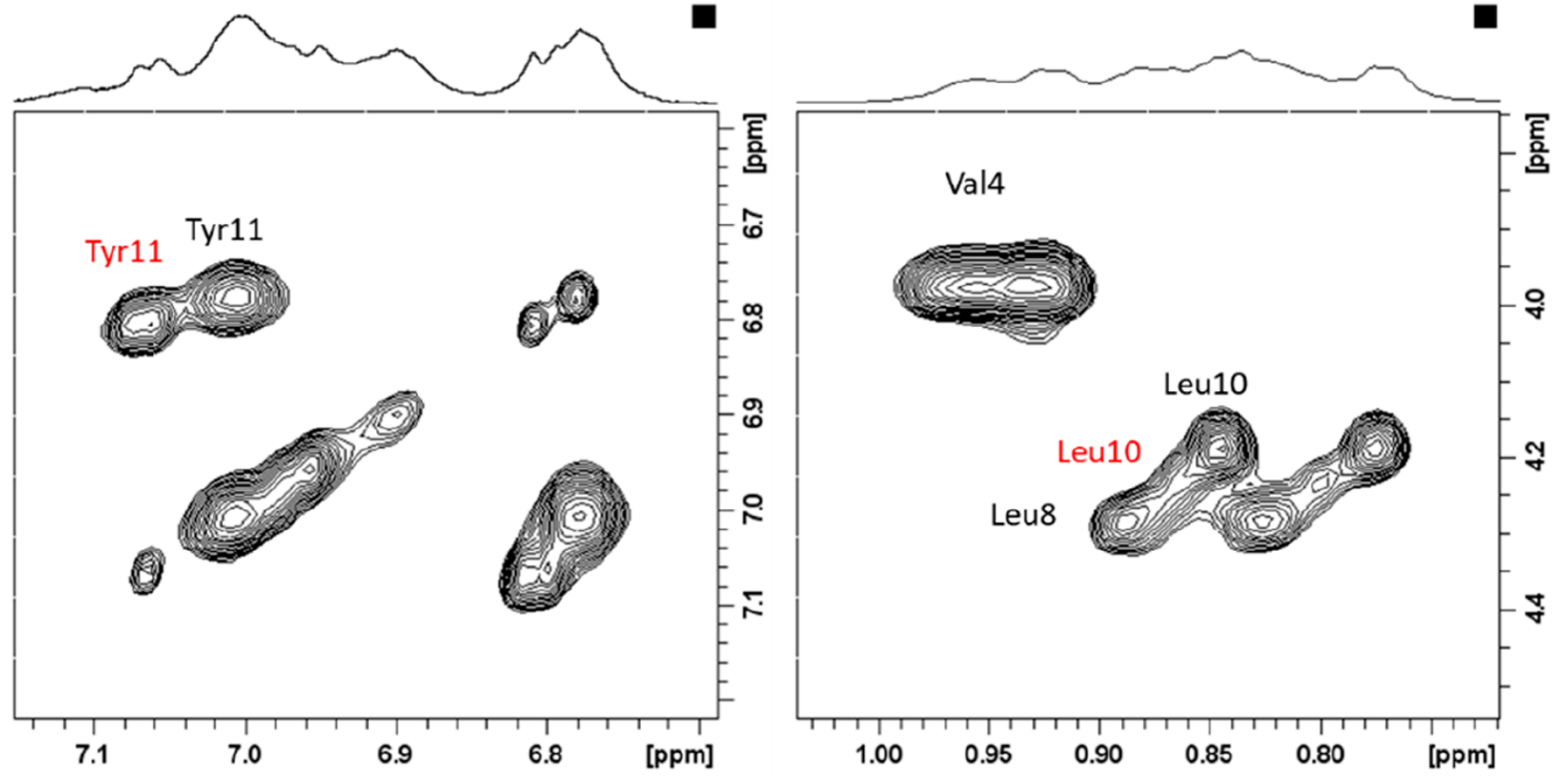
Selected regions of the ^1^H–^1^H TOCSY spectra of 0.8 mM CpaA (Ac-RHEVGHNLGLYHN-NH_2_), pH 7.0, in the presence of 0.75 equiv Zn(II).

Moreover, a careful analysis of 2D TOCSY maps of CpaA-Zn(II) complexes reveals the presence of Leu-10 and Tyr-11 duplicated spin systems, as shown in Figure 11. The existence of two distinct sets of signals belonging to Leu-10 and Tyr-11 is consistent with the occurrence of chemical exchange equilibria between different metal-bound conformations, which is also supported by the large broadening observed on NMR resonances of His and Glu-3. Interestingly one of the two Tyr-11 forms exhibits larger chemical variations upon Zn(II) addition, while the other is completely unchanged (Table 5). Similar behavior is observed for Leu-10, with one of the two forms being more affected than the other. It might be speculated that the two metal-bound forms arise from the possible stabilization effects provided by Tyr-11 phenolate groups (vide infra), thus explaining the different Zn(II)-induced line broadening observed in the AprA systems. The chemical variations upon Zn(II) addition observed on Arg-1, Glu-3, and Tyr-11 may suggest their stabilizing effect on the formed metal complexes. The observed chemical shifts in Arg-1 side chain protons may be explained by the formation of electrostatic interactions between the positively charged guanidine group (−NH_3_^+^) of Arg-1 and the negatively charged carboxyl group (−COO^–^) of Glu-3.^33^ The glutamic acid may be partially exchanged with the water molecule in the coordination sphere at pH 7.0. The second observed stabilization effect, which is suggested by NMR shift variations, is the formation of hydrogen bonding between the hydroxyl group (−OH) of the side chain of Tyr-11 and the amide group (−NH) of the peptide backbone.^56^

## Discussion

**Figure 12:**
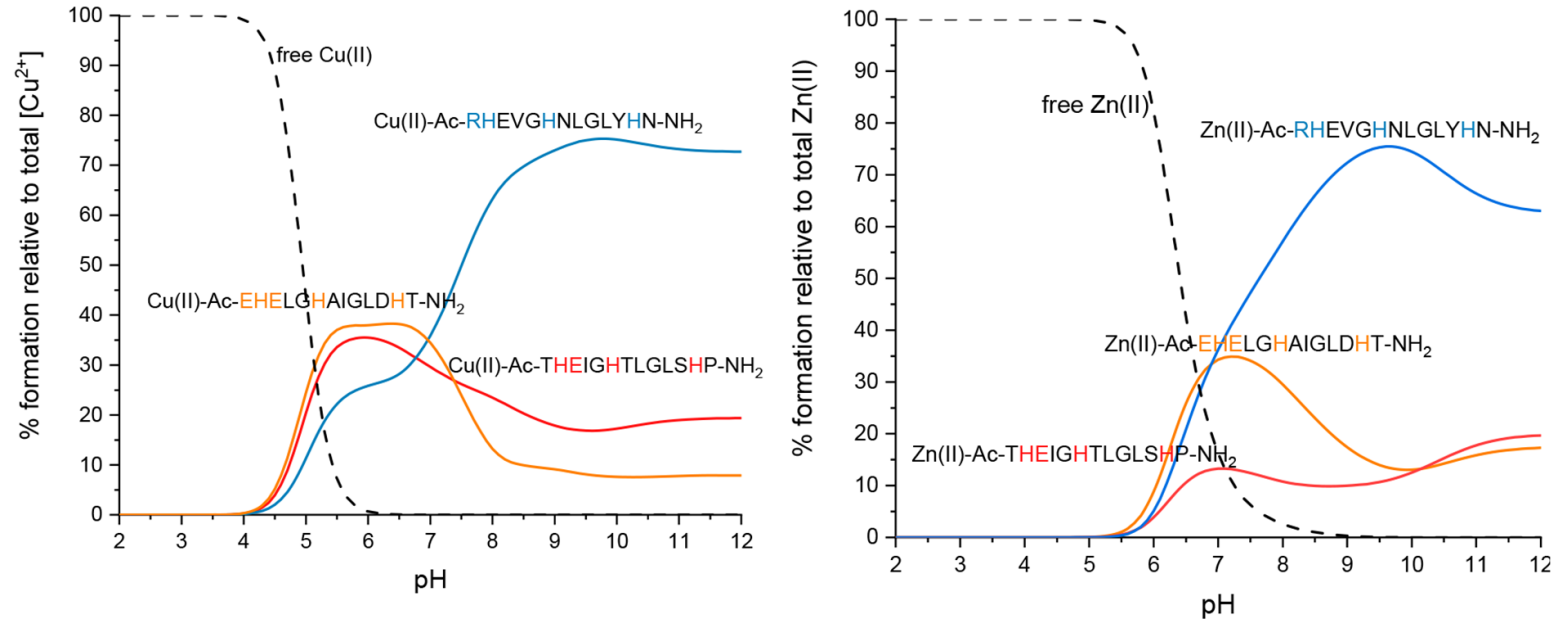
Theoretical competition plots for a solution containing equimolar concentrations (1 mM) of the studied metal-binding domains of MPs and (a) Cu(II) and (b) Zn(II).

In order to determine which metal-binding region of bacterial MPs has the strongest binding affinity for the Zn(II) and Cu(II) metal ions, we simulated a hypothetical scenario in which we mixed equimolar amounts of all of the studied peptides with the metal ion at different pH values (Figure 12). This approach allowed us to directly compare the calculated constants and gain insight into their binding affinity. To further analyze the results, we also compared our findings with those of a previously studied metalloprotease from *Streptococcus pneumonia.*(57)

**Figure 13:**
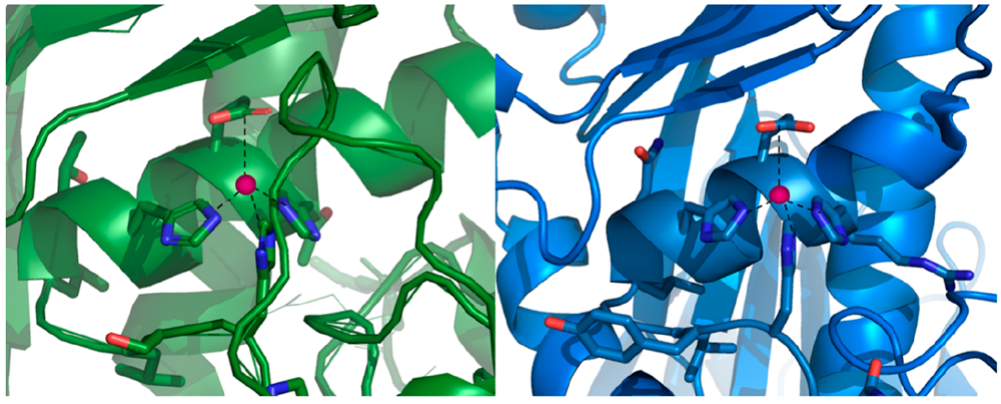
Proposed sketches of the coordination sphere of the Zn(II) complexes of (A) the AprA domain and (B) the CpaA domain at pH 7.4. The structure of the protein is based on simulations by Phyre2. Figures were generated manually using PyMOL.

For both metal ions, Zn(II) and Cu(II), the coordination pattern is similar across a pH range from acidic to around 6.5–7.0 for all studied regions. This pattern consists of three histidine imidazole rings and one oxygen from a carboxylate group of glutamic acid (HExxHxxxxxH) (Figure 13).

The stability of the studied metal complexes was very similar to that of the previously described complex, which confirms the suggested coordination mode. However, the coordination pattern for Cu(II) complexes changes as the pH increases. The binding of one or two deprotonated amide nitrogen atoms begins at pH 6.5, starting with the internal histidine anchoring residue (likely His-6) and progressing toward the N-terminus. This leads to a (1N_im_, 3N^–^) coordination pattern in a square planar geometry above pH 10.

The comparison of the stability of the Cu(II) and Zn(II) complexes for both analyzed domains (Figure S10) is in good agreement with what could have been expected from Irving–William’s series: under equimolar concentration of the two metals and ligand, Cu(II) outcompetes Zn(II) in the whole studied pH range. This result suggests a possible mechanism of inhibition of MPs by copper ions, where copper forms more stable complexes and displaces zinc ions from the binding domain. The lack of copper catalytic activity subsequently leads to the inactivation of the metalloprotease.

Interestingly, even though all the metal complexes exhibit the same coordination mode (3His, 1Glu), the CpaA domain from *Acinetobacter baumanii* has a greater stability above pH 7 for both zinc and copper complexes. This phenomenon could be explained by two factors. The influence of nonbinding amino acids is the first explanation for the increased stability of CpaA complexes with zinc and copper ions. Nonbinding amino acids, which are not directly involved in metal ion coordination, can contribute to the stability of the complex through various interactions such as electrostatic interactions, hydrogen bonding, van der Waals interactions, and solvation effects.^30−33^ The presence of the arginine residue (positively charged amino acid) and the glutamic acid (negatively charged amino acid) in the CpaA region’s sequence contributes to the formation of salt bridges.^33,58,54^ These electrostatic interactions can occur between the acidic side chains of glutamic acid and the basic side chains of arginine in a pH range from 4.5 to 12 when both the carboxyl group is deprotonated (−COO^–^) and the guanidine group is protonated (−NH_3_^+^), which may partly explain the greater stabilization in comparison to the AprA domain. Since peptides can accumulate various charged or polar amino acid side chains, the formation of intramolecular hydrogen bonds is a common feature of metal–peptide complexes.^31,33,58^ The influence of tyrosine at a basic pH is the next stabilizing factor of the Metal-Ac-RHEVGHLNLYHN-NH_2_ complex. The side chain of tyrosine contains a hydroxyl group (−OH) on the phenyl ring that can form hydrogen-bonding interactions with the amide groups (−NH) of the peptide backbone, which can enhance the stabilization of the complex.^56^ The NMR results of the Zn(II)-CpaA system suggest a stabilizing effect of tyrosine. The greater thermodynamic stability of CpaA metal complexes can also be explained by the formation of specified secondary structures. Defined conformation features can play an important role in the stabilization of metal complexes by forming hydrogen bonds.^59^ These hydrogen bonds can significantly enhance the binding affinity and thermodynamic stability of the complex by providing multiple binding sites for the metal ion and restricting the conformational mobility of the metal ion within the complex.^30^ The structure of zinc complexes with the CpaA domain and AprA is primarily unstructured, with a slight inclination toward helical structure, depending on the pH values, as observed through CD spectra analysis (Figures S7 and S9). Furthermore, copper complexes with the AprA metal-binding domain exhibit an undefined structure with a mixture of the random coil and the α-helix at acidic pH (Figure S2). However, as the pH increases, the complex adopts an α-helical structure, thus stabilizing the metal complex. Notably, the Cu(II)-CpaA complex adopts an unusual PPII conformational feature at pH 7–9 (Figure 5). The PPII helix, which consists of a left-handed extended helical conformation, is relatively rare in proteins and has mostly been observed in small peptides and regions of some proteins.^52^ PPII helices are often stabilized by main-chain–water hydrogen bonds.^60^ They have been shown to play a crucial role in the stability and function of various proteins, such as regulating enzymatic activity and ligand binding. Furthermore, the extended nature of the PPII helix plays an important role in conformational changes, resulting in the formation of amyloid fibrils by the prion protein or amyloidogenic lysozyme.^61^ As we precisely described in the previous sections, the PPII structure of the Cu(II)-CpaA complex is stabilized by electrostatic interactions between positively charged Arg-1 and negatively charged Glu-3 at pH 7–9 (observed in the far-UV CD spectra in Figure 5). Moreover, this phenomenon can be observed only in the case of copper complexes above pH 6.5 due to the exchange in the coordination sphere from the oxygen from glutamic acid to nitrogen from the amide of the peptide backbone. In contrast, zinc is not able to coordinate amide nitrogen and thus leaves glutamic acid in a coordination sphere, preventing the formation of salt bridges. This is also confirmed by the NMR results of the Zn(II)-CpaA system, in which shifts of Hα-Hγ correlation signals of Glu-3 are still observed at pH 7.0 (Figure 10B). However, as the pH increases, the water molecule deprotonates and binds to the zinc ion. The second possible explanation for why copper complexes can form the PPII structure while zinc complexes cannot is the differences in the geometries of formed metal complexes. Copper, which at alkaline pH coordinates the nitrogen from the peptide backbone, forms complexes with a square planar structure, which is confirmed by UV–vis results (Figure S5A). The planar geometry of the formed copper complexes enables a spherical arrangement of amino acids in the form of an extended PPII structure. In contrast, the tetrahedral structure of the zinc complexes may limit the range of ligand orientations and therefore restrict the formation of the PPII structure.

How can the copper-induced changes in the structure of the catalytic domain of MPs affect their enzymatic activity? The most common secondary structure of the active site of bacterial metalloproteases is divided into two segments: the first half of the zinc-binding motif, HExxH, adopts the α-helix conformation, while the polypeptide chain at the end of the domain takes a turn, which is mediated by the glycine of the amino acid sequence.^62,63^ Moreover, the secondary structure prediction of one of the bacterial MP families revealed that the analyzed domains are dominated by 41.64% of random coils along with 32.12% of α-helices, while 20.36% of residues were in extended sheet.^64^ This prediction is in good agreement with our results (Figures S7 and S9). A change in the secondary structure of the catalytic site to a PPII helix can significantly impact the enzymatic activity of a metalloprotease. The PPII helices are characterized by a more extended and less compact structure than the other secondary structures. The higher degree of flexibility can affect the positioning and orientation of the catalytic residues and the substrate within the active site. This could result in a loss of coordination of the catalytic metal ion and/or mispositioning of the substrate, leading to an inhibition of the enzyme’s activity. Additionally, a change in the secondary structure could also affect the stability and folding of the protein. Misfold could lead to the formation of aggregates, which could prevent the proper formation of the active site and lead to a loss of activity.

The results obtained in this study confirm the ability of copper ions to inhibit MPs in a metal-based manner. Copper ions are able to displace Zn(II) from their binding site in the catalytic domain across a wide range of pH values due to the formation of thermodynamically more stable complexes within the domain. The Cu(II) complexes are characterized not only by a different coordination geometry but also changes in the secondary structure of the catalytic domain induced by the copper ion. Those precisely described factors may lead to inhibition of the enzymatic activity of metalloproteases.

### Competition Study Imitating In Vivo Conditions

Adequate concentrations of trace metal ions must be maintained to ensure proper protein function and avoid toxic effects. The relative concentration of particulate metals in relation to other metal ions within the environment profoundly impacts bacterial physiology, as these metals compete for binding sites within proteins.^65^ How does this affect the activity of the AprA and CpaA metalloproteases? AprA, a MP produced by *P. aeruginosa*, is localized within the extracellular environment. This secretion enables AprA to interact with the host’s proteins, initiating their enzymatic degradation.^25^*P. aeruginosa* is characterized as an opportunistic pathogen, and infections pose a pronounced threat to individuals afflicted by cystic fibrosis (CF). Microbes colonize the airway mucus of patients with cystic fibrosis (CF), where they compete for nutrients, such as metals, with host cells and other microbes. It was reported in the literature that in CF sputum, *P. aeruginosa* increases the expression of proteins involved in zinc uptake and regulation. Moreover, multiple studies have demonstrated that zinc ion chelation inhibits the activity of extracellular MPs, which directly affects the colonization of *P. aureginosa*.^66^ In light of these findings, in this work, we projected the competitive study between Zn(II) and Cu(II) of the metal-binding sites of AprA under conditions imitating an *in vivo* environment. For this purpose, we relied on reports of metal concentrations and the pH of infected tissues. In the CF sputum, zinc ions outweigh copper at a ratio of Zn(II)/Cu(II) = 10:1,^67^ and the pH of the CF tends to be more acidic compared to the physiological pH, hovering around 7.00.^68,69^

*A. baumannii* is the causative agent of a broad range of diseases including pneumonia, bacteremia, urinary tract infections, and meningitis. Moreover, its ventilator-associated pneumonia affects many intensive care units.^70^ As in the case of *P. aureginosa*, it has been reported that chelation of zinc ions exhibits inhibitory effects against *A. baumannii* infections.^71^ CpaA, like numerous other metalloproteases, is localized predominantly in extracellular environment.^27^

**Figure 14:**
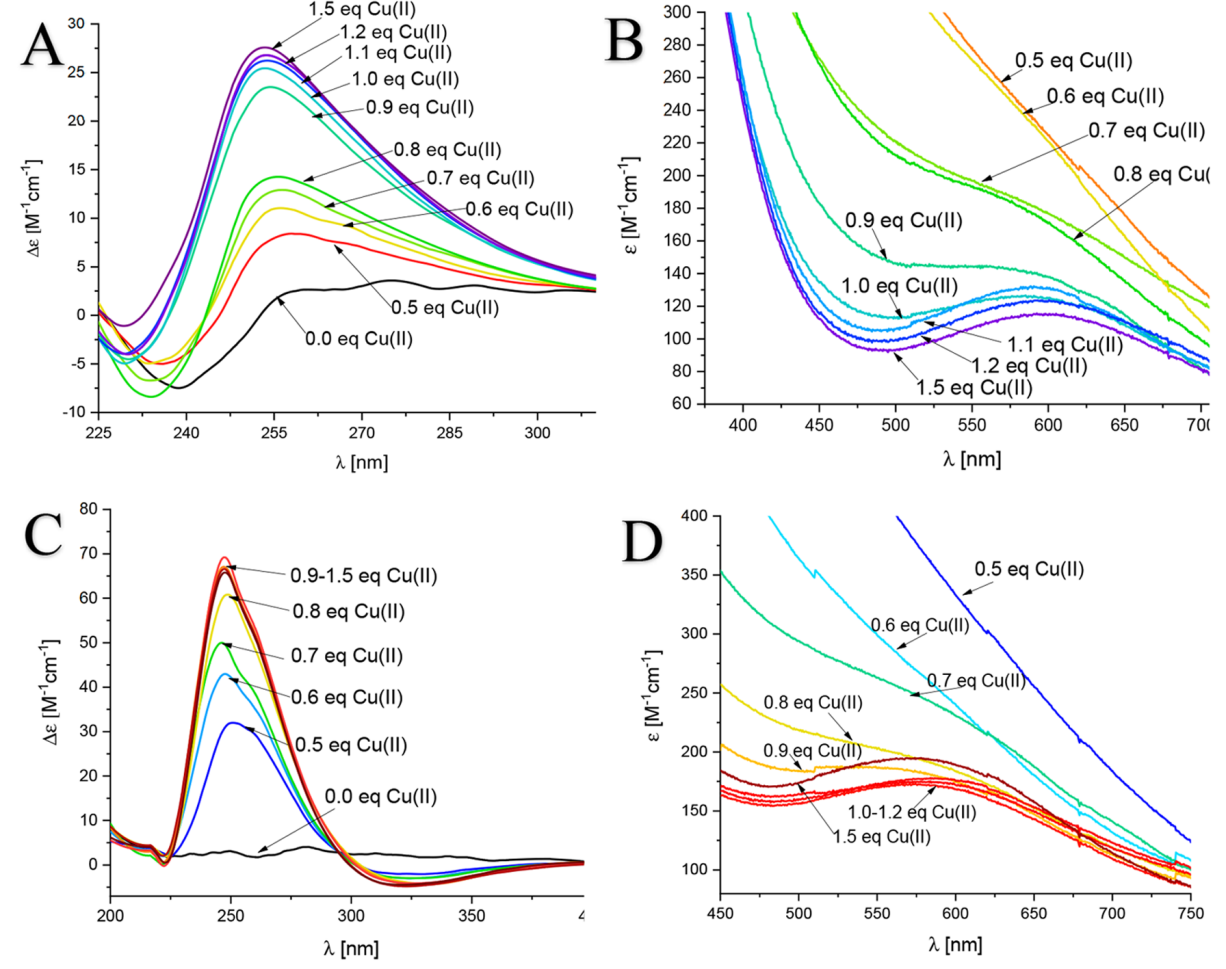
(A and C) CD and (B and D) visible spectra of (A and B) the Zn(II)-AprA complex and (C and D) the Zn(II)-CpaA complex with the increasing addition of equivalents of Cu(II) at pH 7.0 under simulated *in vivo* conditions. The initial peptide/Zn(II)/Cu(II) ratio is 1:10:0.

Considering this, our simulation of the in vivo conditions for competitive study in the case of the CpaA protein involves emulating the lung-tissue environment, given *A. baumannii’s* involvement in pneumonia. A similar concentration ratio has been observed in infected lung tissue, mirroring that of CF sputum: the Cu(II)/Zn(II) ratio is 1:10.^71,72^ Bacterial infections, which lead to the inflammation, create the microenvironment of the lungs with a pH around 7.2–7.4.^73^ As shown in Figure 14A and B, during the CD/visible spectroscopic titration of copper ions into the solution of the Zn(II)-AprA complex, we can observe the stepwise formation of the Cu(II)-AprA complex, which is completed at the ratio of AprA/Zn(II)/Cu(II) = 1:10:1. During the experiment, we can observe the characteristic bands for the Cu(II)-AprA complexes, in particular those at 255 nm in the CD spectra and 600 nm in the visible spectra. The bands correspond to the copper complexes with the AprA model at pH 7, as we precisely described in the previous section. Additional amounts of copper do not influence the bands in the visible/CD spectra, which confirms that the binding of copper has ended at the previously mentioned molar ratio of 1:10:1 AprA/Zn(II)/Cu(II). Similar results have been obtained for the peptide model of CpaA from *A. baumanii*. In the case of titration by Cu(II) of the Zn(II)-CpaA complex, the substitution of zinc by copper ions is completed at the CpaA molar ratio of CpaA/Zn(II)/Cu(II) = 1:10:1. In the visible and CD spectra of the CpaA-Zn(II)-Cu(II) system (Figure 14C and D), bands corresponding to the Cu(II)-CpaA complexes at 600 nm in the visible spectrum and 250 nm in the CD spectrum increasing during the addition of copper ions to the solution. These results confirm that Cu(II) is able to displace Zn(II) from its binding site under simulated in vivo conditions, even during a 10-fold excess of zinc ions, as in the infected tissues. However, this is only preliminary research that shows the capacity of Cu(II) ions as a supplementary mechanism for improving the antibacterial strategy against bacterial MP. To fully realize its inhibitory function, additional chemical compounds are required. Interestingly, copper has already been tested alongside antibiotics, resulting in synergistic effects with antimicrobial agents like gatifloxacin, capreomycin, and disulfiram. These findings offer an avenue for prospective investigations.^74^

## Conclusions

Metalloproteases are a class of extracellular proteolytic enzymes that are commonly found among primary and opportunistic pathogens. These enzymes play a crucial role in the colonization and infection processes of microorganisms, making them attractive targets for the development of new antibiotics. This study performed a detailed analysis of the metal-binding regions of two MPs from multidrug resistant bacteria, AprA from *P. Aureginosa* and CpaA from *A. baumanii.* Our work identified coordination patterns of the metal complexes consisting of three histidine imidazole rings and one oxygen from a carboxylate group of glutamic acid (HExxHxxxxxH) for all the studied regions below pH 7. The coordination pattern for Cu(II) complexes changes as the pH increases, as copper coordinates with nitrogens from the peptide backbone. Furthermore, the study found that both AprA and CpaA have a significantly higher affinity for Cu(II) compared to Zn(II). This observation may provide an explanation for the mechanism of inhibition of MPs by copper ions, as it suggests a potential for copper to displace zinc ions from the binding domain and inhibit the enzymatic activity of the MPs, as previously described in the literature.^57,75^ The results of this analysis led also to the conclusion that the CpaA domain has greater stability above pH 7 for both zinc and copper complexes compared to the AprA metalloprotease. The increased stability of the CpaA domain can be attributed to the presence of specific nonbinding amino acids and their intramolecular interactions. Tyrosine, which contains a hydroxyl group (−OH) on the side chain, forms hydrogen-bonding interactions with the peptide backbone. Arginine in the first position (favorable for PPII structure) forms hydrogen bonding interactions with glutamic acid, which enhance the stability of the metal complex and contribute to the formation of a rare protein secondary structure known as PPII. Moreover, even nonpolar hydrophobic amino acids such as leucine, which occurs in the center of the peptide sequence of the CpaA domain, have an impact in the formation of the PPII structure due to trans-peptide bond conformation. PPII is known to play a crucial role in the stability and function of various proteins, providing insight into the potential mechanisms of MPs inhibition. The results of this peptide-based study push us toward the need to hypothesize about the effective role of metal-based inhibitors. Peptides derived from the metal/ligand binding sites of proteins serve as informative protein-mimetics models and are commonly used in drug design to determine the mechanism of metal/inhibitor–protein binding.^76,77^ However, the investigations on peptide models are limited in their ability to explain biological phenomena, since they do not take into consideration second-shell interactions or interactions with regions from other parts of the discussed proteins. Therefore, to gain a more comprehensive understanding of the role of Cu(II) as an additional MP inhibitor, future research exploring the interactions of the entire metalloprotease with Cu(II) and other agents against MPs is highly recommended. This broader approach will shed light on how metal-based inhibitors may function in a real biological environment and provide valuable insights into the development of targeted therapies.

## Supporting Information Available

Complementary mass spectra; EPR parameters; proton NMR assignments; competition plot; and UV–vis, CD, and EPR spectra (PDF)
