# Supplementary material for: Copper Forms a PPII Helix-Like Structure with the Catalytic Domains of Bacterial Zinc Metalloproteases
Source: Inorg Chem. 2023 Nov 1;62(45):18425–39. doi: 10.1021/acs.inorgchem.3c02391 (PMC10647932; doi:10.1021/acs.inorgchem.3c02391)
Supplement: Supplementary file 1 — ic3c02391_si_001.pdf [file ic3c02391_si_001.pdf]

Supporting Information

Copper forms a PPII helix-like structure with the catalytic domains of bacterial zinc metalloproteases

Paulina Potok,[1] Arian Kola,[2] Daniela Valensin,[2] Merce Capdevila,[3] Sławomir Potocki[1*]
slawomir.potocki@uwr.edu.pl

[1] Faculty of Chemistry, University of Wroclaw, 50-383 Wroclaw, Poland
[2] Department of Biotechnology, Chemistry and Pharmacy, University of Siena, Via A. Moro 2, 53100 Siena, Italy
[3] Departament de Química, Universitat Autònoma de Barcelona, 08193 Cerdanyola del Vallès, Spain

Table S1. EPR parameters of Cu(II)-AprA (Ac-THEIGHTLGLSHP-NH_2) complex with number of proposed nitrogen donors. The given parameters were simulated using WinEPR SimFonia Version 1.2 (Bil lerica, USA). Nuclear spin of copper: 3/2; nuclear spin of nitrogen: 1; MW Frequency 9.6195-9.6260 GHz

pH	$A_{\|\|}$ [G] (A_{zz})	$g_{\|\|}$ (g_z)	$g_{\perp}$ (g_x=g_y)	Proposed donors
3.13	122.1	2.42	2.08	0N
4.03	120.0	2.41	2.07	0N
5.10	155.5	2.30	2.06	1N
6.03	162.5	2.28	2.05	2N or 3N
7.06	164.5	2.24	2.05	2N or 3N
8.07	163.5	2.24	2.05	2N or 3N
9.12	165.7	2.24	2.05	3N
10.01	185.7	2.19	2.04	3N or 4N

Table S2. EPR parameters of Cu(II)-CpaA (Ac-RHEVGHNLGLYHN-NH_2) complex with a number of proposed nitrogen donors. The given parameters were simulated using WinEPR SimFonia Version 1.2 (Bille rica,USA). Nuclear spin of copper: 3/2; nuclear spin of nitrogen: 1; MW Frequency 9.6195-9.6260 GHz

pH	$A_{\|\|}$ [G] (A_{zz})	$g_{\|\|}$ (g_z)	$g_{\perp}$ (g_x=g_y)	Proposed donors
3.05	120.1	2.42	2.06	0N
3.96	119.0	2.42	2.05	0N
5.01	168.3	2.30	2.05	2N
6.05	168.2	2.29	2.05	2N or 3N
7.10	178.0	2.25	2.05	3N
8.09	181.4	2.23	2.04	3N or 4N
9.09	186.4	2.20	2.04	3N or 4N
10.01	201.7	2.19	2.04	4N
11.00	209.5	2.19	2.04	4N

Table S3. Proton NMR assignment of AprA (Ac-THEIGHTLGLSHP-NH_2) 0.8mM pH 7,04 T=298K

Residue	NH	Hα	$H\beta_1$	$H\beta_2$	$H\gamma_1$	$H\gamma_2$	$H\delta_1$	$H\delta_2$	H4	H2
Ac		2,09								
Thr1	8,18	4,31	4,20		1,17					

His2		4,64	3,12						7,00	7,83
Glu3	8,27	4,28	1,99	1,87	2,16					
Ile4	8,23	4,15	1,87		1,48	1,21	0,90			
Gly5	8,48	3,94								
His6		4,69	3,12						7,00	7,83
Thr7	8,18	4,23	4,17		1,17					
Leu8	8,37	4,34	1,62				0,90			
Gly9	8,39	3,94								
Leu10	8,04	4,39	1,59				0,90			
Ser11	8,28	4,44	3,82							
His12		4,90	3,11	3,02					7,00	7,84
Pro13		4,39	2,28	1,99			3,75	3,49		
NH2		7,06	7,68							

Table S4. Proton NMR assignment of CpaA (Ac-RHEVGHNLGLYHN-NH_2) 0.8mM pH 7,04 T=298K

**Residue**	**NH**	**Hα**	**Hβ$_1$**	**Hβ$_2$**	**Hγ$_1$**	**Hδ$_1$**	**H4**	**H2**
Ac		2,02						
Arg1	8,27	4,23	1,75	1,68	1,56	3,18		
His2*		4,54	3,12	3,02			6,95	7,74
Glu3	8,38	4,32	2,00	1,86	2,17			
Val4	8,21	4,10	2,08		0,93			
Gly5	8,49	3,92						
His6*		4,58	3,05	2,89			6,95	7,74
Asn7		4,65	2,82	2,72				
Leu8	8,32	4,30	1,67	1,59		0,86		
Gly9	8,38	3,90						
Leu10	7,88	4,24	1,43	1,34		0,86		
Tyr11	8,11	4,56	3,06	2,90			(2,6H)<br>7,08	(3,5H)<br>6,81
His12*		4,60	3,08	2,99			6,95	7,74
Asn13		4,62	2,78	2,71				

*These assignments can be inter-exchanged

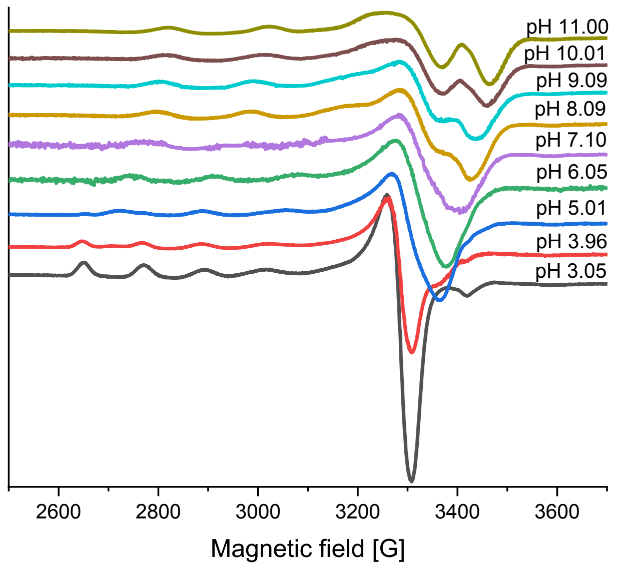

Figure S1. EPR spectra performed for the Cu(II)-AprA (Ac-THEIGHTLGLSHP-NH_2) complex, 3.00–11.00 pH range.

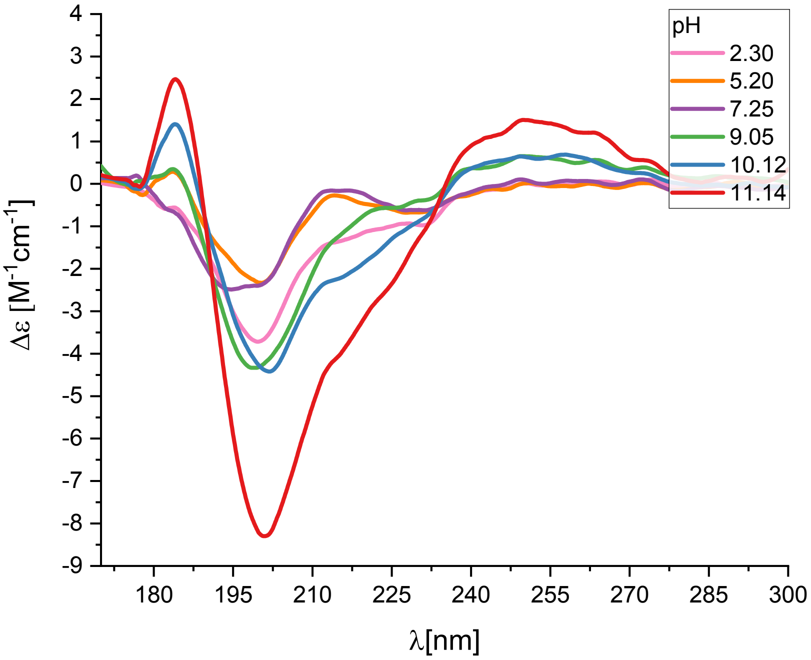

Figure S2. The far-UV CD spectra for the Cu(II)-AprA (Ac-THEIGHTLGLSHP-NH_2) complex, at different pH values at a 1.0:1.1 Cu(II)/peptide ratio in aqueous solution.

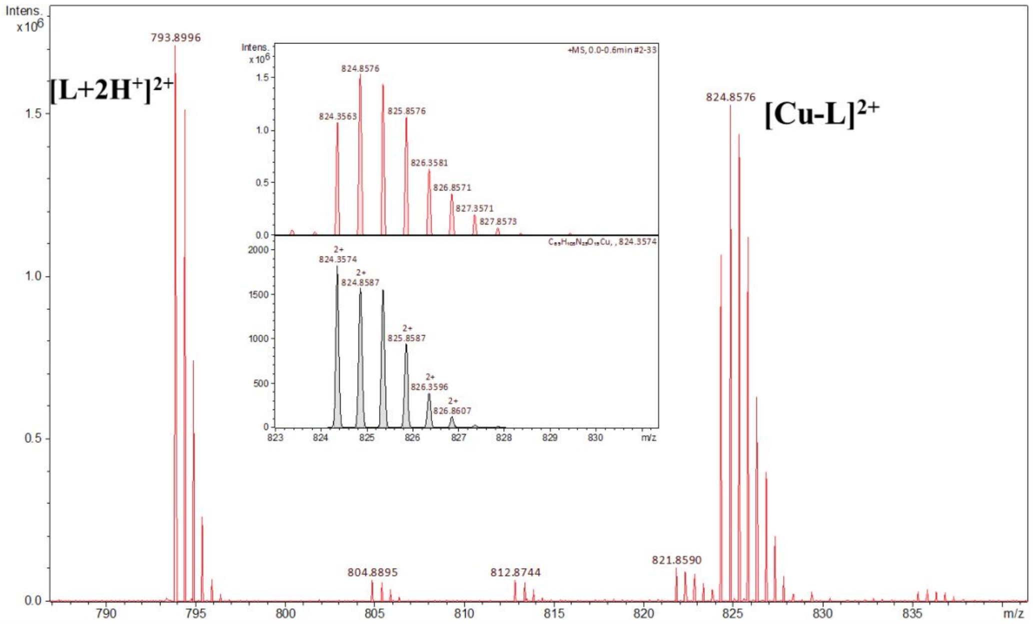

Figure S3. ESI-MS spectrum of a system composed of the CpaA (Ac-RHEVGHNLGLYHN-NH_2) ligand (L) and Cu(II) ions in the range of *m*/*z* 785–840 at pH 7.0 (1:1 M:L). In the middle, the simulated and experimental isotopic distribution spectra with a peak at *m*/*z* 824.86 are presented.

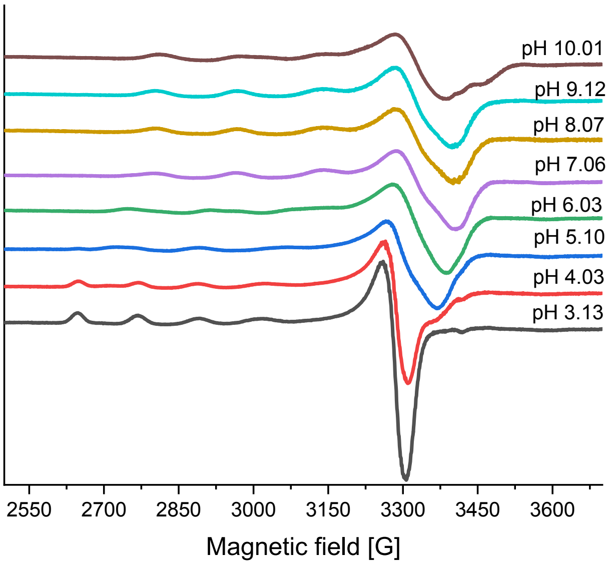

Figure S4. EPR spectra were performed for the complex Cu(II)-CpaA (Ac-RHEVGHNLGLYHN-NH_2), 3.00–11.00 pH range.

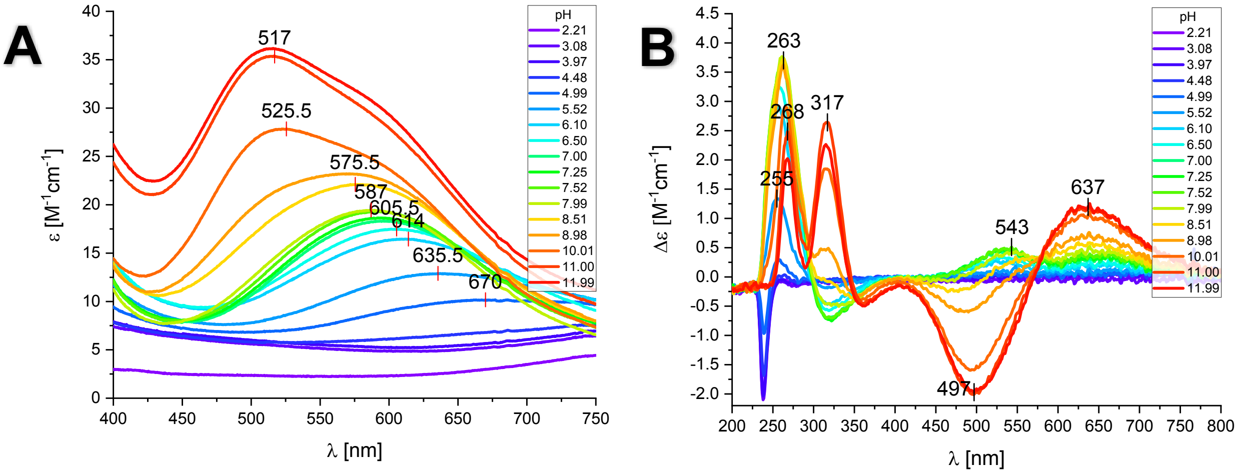

Figure S5. Vis absorption spectra (A) and CD spectra (B) of Cu(II)-CpaA (Ac-RHEVGHNLGLYHN-NH_2) at different pH values; at different pH values at a 1.0:1.1 Cu(II)/peptide ratio in aqueous solution. The wavelength of maximum absorption is reported for each spectrum.

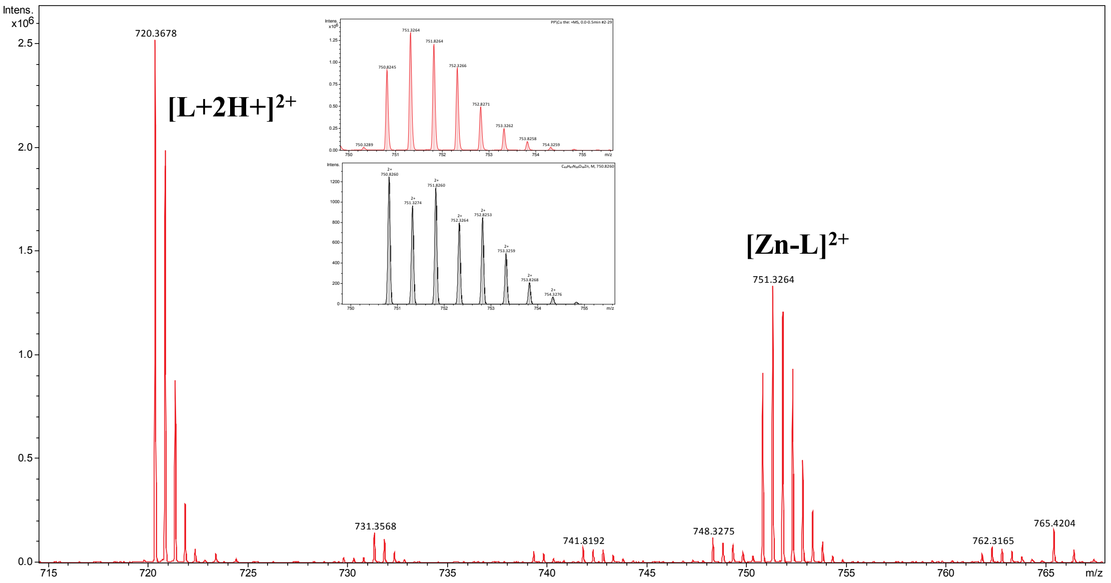

Figure S6. ESI-MS spectrum of a system composed of the AprA (Ac-THEIGHTLGLSHP-NH_2) ligand and Zn(II) ions in the range of *m/z* 715–770 at pH 7.0 (1:1 M:L). In the middle, the simulated and experimental isotopic distribution spectra with a peak at *m/z* 751.33 are presented.

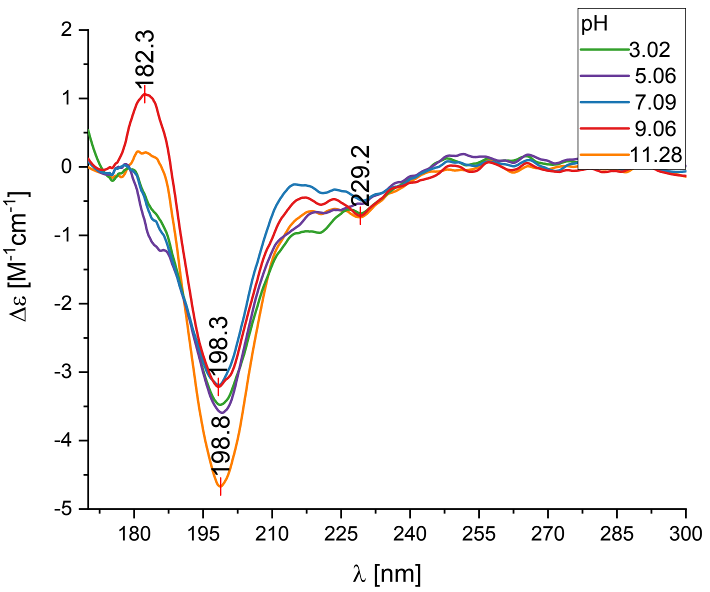

Figure S7. The far-UV CD spectra for the Zn(II)-AprA (Ac-THEIGHTLGLSHP-NH_2) complex, at different pH values at a 1.0:1.1 Zn(II)/peptide ratio in aqueous solution.

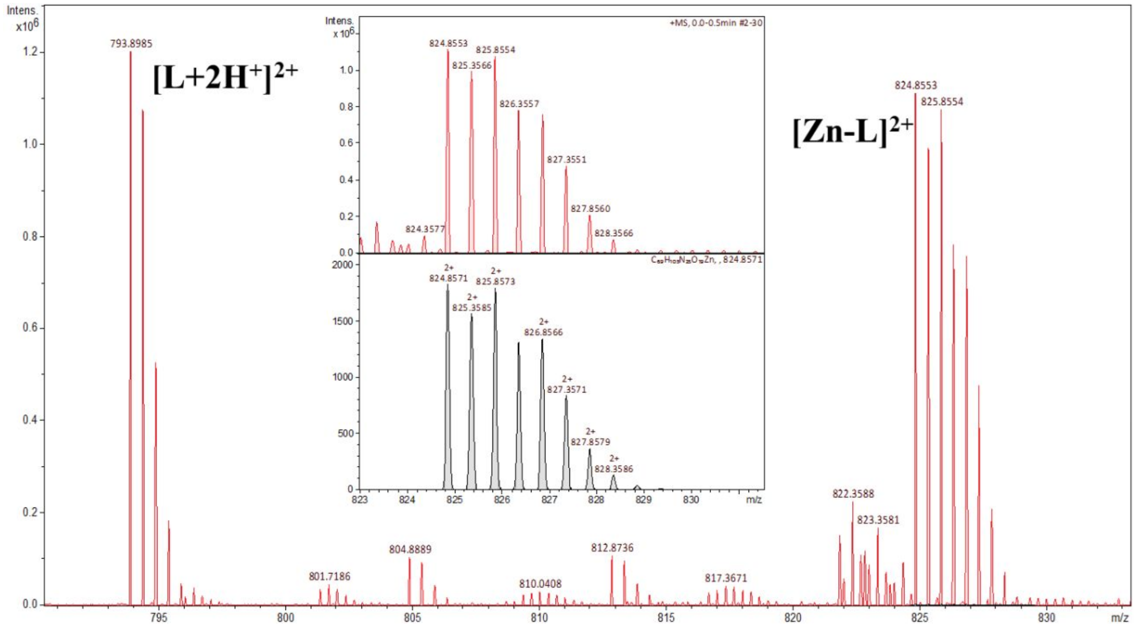

Figure S8. ESI-MS spectrum of a system composed of the CpaA (Ac-RHEVGHNLGLYHN-NH_2) ligand (L) and Zn(II) ions in the range of *m*/*z* 790-835 (1:1 M:L). In the middle, the simulated and experimental isotopic distribution spectra with a peak at *m*/*z* 824.86 are presented.

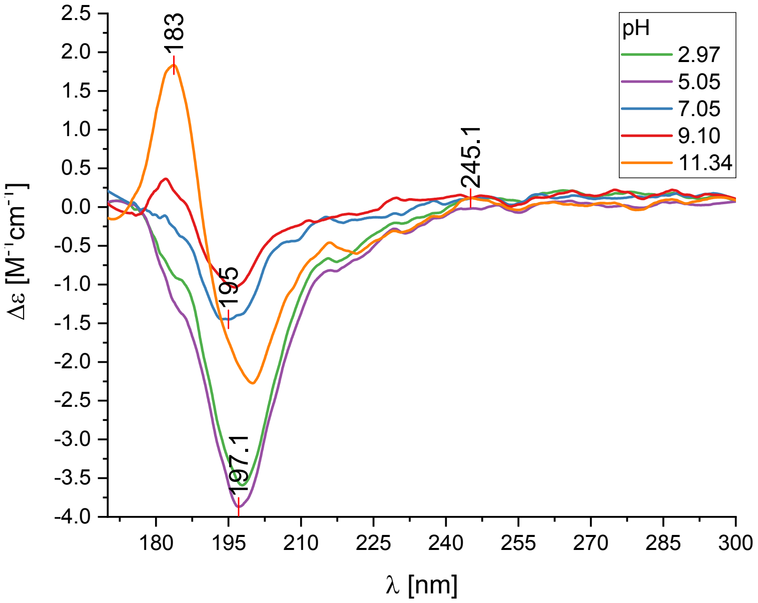

Figure S9. The far-UV CD spectra for the Zn(II)-CpaA (Ac-RHEVGHNLGLYHN-NH_2) complex, at different pH values at a 1.0:1.1 Zn(II)/peptide ratio in aqueous solution.

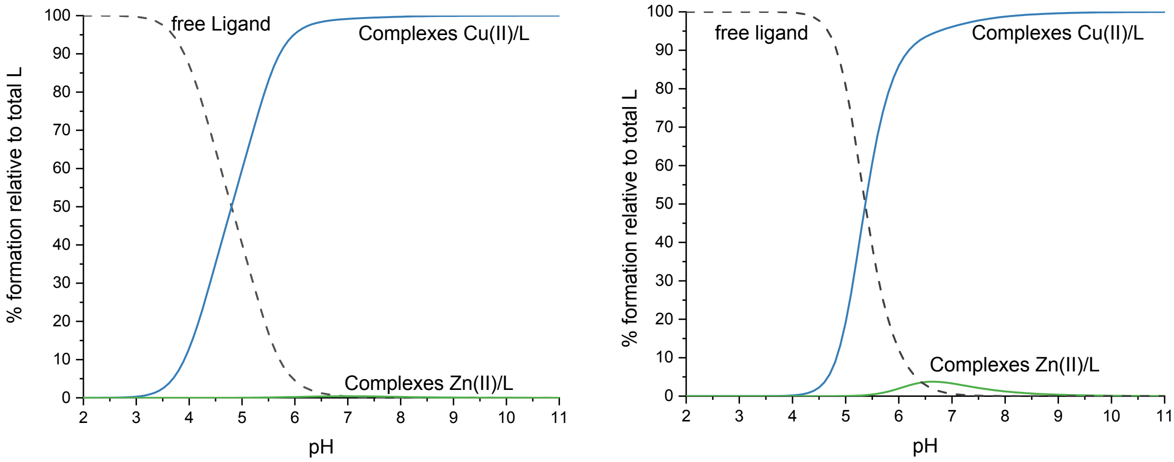

Figure S10. Theoretical competition plots showing the comparison of thermodynamic stability between the complexes of Zn(II), and Cu(II) ions with the ligand L: (A) AprA Ac-THEIGHTLGLSHP-NH_2 (B) CpaA Ac-RHEVGHNLGLYHN-NH_2 peptide. The molar ratio for the ligand and metal ions L:Zn(II):Cu(II) is 1:1:1.
